# Selective glycoprotein detection through covalent templating and allosteric *click*-imprinting†
†Electronic supplementary information (ESI) available: Synthetic procedures, synthesis of **DFC**, NMR spectra, surface modification and characterisation in addition to SPR analysis. See DOI: 10.1039/c5sc02031j



**DOI:** 10.1039/c5sc02031j

**Published:** 2015-06-17

**Authors:** Alexander Stephenson-Brown, Aaron L. Acton, Jon A. Preece, John S. Fossey, Paula M. Mendes

**Affiliations:** a School of Chemical Engineering , University of Birmingham , Edgbaston , Birmingham , West Midlands B15 2TT , UK . Email: p.m.mendes@bham.ac.uk; b School of Chemistry , University of Birmingham , Edgbaston , Birmingham , West Midlands B15 2TT , UK . Email: j.s.fossey@bham.ac.uk

## Abstract

Many glycoproteins are intimately linked to the onset and progression of numerous heritable or acquired diseases of humans, including cancer. Indeed the recognition of specific glycoproteins remains a significant challenge in analytical method and diagnostic development. Herein, a hierarchical bottom-up route exploiting reversible covalent interactions with boronic acids and so-called click chemistry for the fabrication of glycoprotein selective surfaces that surmount current antibody constraints is described. The self-assembled and imprinted surfaces, containing specific glycoprotein molecular recognition nanocavities, confer high binding affinities, nanomolar sensitivity, exceptional glycoprotein specificity and selectivity with as high as 30 fold selectivity for prostate specific antigen (PSA) over other glycoproteins. This synthetic, robust and highly selective recognition platform can be used in complex biological media and be recycled multiple times with no performance decrement.

## Abstract 2

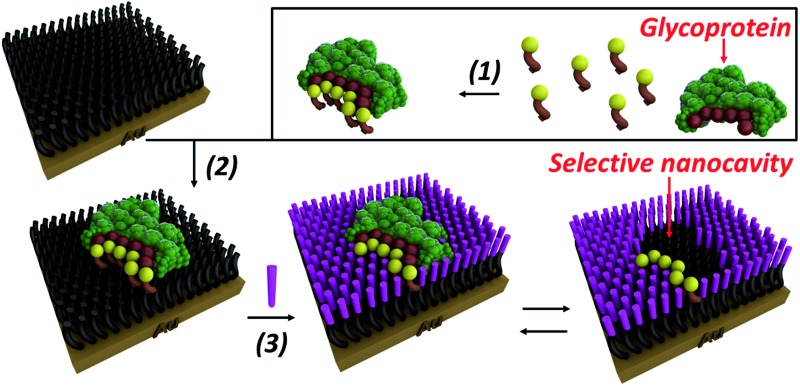
A hierarchical bottom-up route exploiting reversible covalent interactions with boronic acids and so-called click chemistry for selective glycoprotein detection is described. The self-assembled and imprinted surfaces confer high binding affinities, nanomolar sensitivity, exceptional glycoprotein specificity and selectivity.

## Introduction

Antibodies are widely used as receptor sites in the detection, quantification and purification of many proteins including clinically relevant glycoproteins.^1^ However, antibodies suffer from poor stability, need special handling and require a complicated, costly production procedure. Furthermore, the peculiarities of intracellular machinery, which is utilised in the commercial production of antibodies, is not ideally suited for the production of high affinity antibodies against carbohydrate-based antigens.^2,3^ For all of these reasons, more robust synthetic alternatives are sought.

Molecular imprinting is a template directed process,^4^ where polymer networks are formed around molecular structures of interest, literally producing a molecular mould. In this way, artificial binding sites can be produced and used in a number of settings, including chromatographic separation,^5,6^ sensors,^7^ catalysis^8^ and drug delivery.^9^ Whilst this approach has been successfully applied to small molecule recognition, intrinsic limitations of traditional molecular imprinting mean it is less suitable for larger, multi-recognition domain molecules, such as glycoproteins. Specifically, key issues include entrapment within the network, poor re-binding kinetics and heterogeneity in binding pocket affinity.^10,11^ Herein, a synthetic recognition platform based on self-assembly approaches and molecular imprinting concepts that exhibits antibody-like behaviour and exceptionally high selectivity for target glycoproteins is described.

In order to provide glycoprotein specificity, boronic acid (BA)-based carbohydrate receptors were selected as an appropriate binding motif for glycoprotein recognition. BAs are ideal candidates for effective formation of glycoprotein recognition sites because they covalently and reversibly bind carbohydrates to form five- or six-membered cyclic boronic esters in aqueous alkaline solution, while the cyclic esters dissociate when the medium is changed to acidic pH.^12–17^ Thus, it permits template removal (no entrapment) and continuous analyte monitoring through reversible binding.

**Fig. 1:**
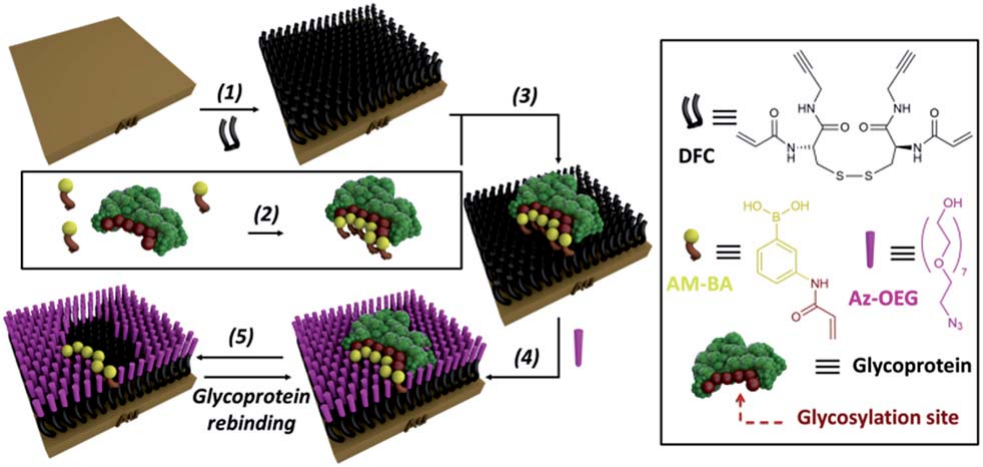
Experimental design for formation of surface restricted click-imprinted binding sites for glycoproteins.

In order to overcome the inherent problems of polymer imprinted receptors, an open, pseudo 2D, recognition domain was envisaged that, as previously demonstrated, allows for excellent mass transfer of proteins into and out of imprinted sites.^18^ Open receptor pockets were constructed from a hierarchical, highly predicable and controllable approach (Fig. 1).

Key to achieving glycoprotein selectivity was decorating the open pocket with suitably positioned BAs, *i.e.* BAs must be presented in the ideal orientation for interaction with specific saccharide fragments within the binding pocket. In contrast with previous work where BAs have been used as anchors to attach glycoproteins to surfaces,^19,20^ a pre-templating approach was employed to achieve the optimal combination of allosteric imprint and reversible covalent binding *via* BAs. An acrylamide appended BA was mixed in solution with a glycoprotein which would find the ideal, strongest affinity, binding sites (by forming boronic esters between the saccharide fragments and the BAs). Now the glycoprotein, bound by ideally positioned BAs bearing functional acrylamides, is bound to a complementarily pre-treated surface, and the empty space on the surface is *capped off* with otherwise inert functionality and the glycoprotein removed. This processes leaves behind a binding site that matches both the shape and the very specific orientations of saccharide fragment through a unique pseudo 2D allosteric and covalent complementarity.

## Results and discussion

A self-assembled monolayer (SAM) on a gold surface of an orthogonally functionalised acrylamide–alkyne cysteine derivative was prepared from its corresponding disulphide dimer (**DFC**, Fig. 1). Orthogonal functionalisation allows surface components to follow two possible pathways: (i) the acrylamide part can engage in polymerisation activity with acrylamide units of the template glycoprotein and each other, fixing the surface geometry; and (ii) the alkyne units can undergo copper catalysed azide alkyne cycloaddition (CuAAC) reactions (so-called *click* reaction),^21^ which can be used to *cap off* residual alkyne functionality and build an ordered pocket around the bound template, thus delivering the *click*-imprinted pocket.

The construction of the sensing sites that mimic antibody in one sense but surpass them by offering unique reversible covalent recognition arrays, on surfaces involves five major steps as outlined in Fig. 1. **DFC** SAMs are prepared by immersing clean gold substrates in 0.1 mM methanolic solutions of **DFC** for 24 hours (step 1). **DFC** was synthesised from commercially available doubly Boc protected cystine, (Boc-Cys-OH)_2_. The alkyne and acrylamide cross-linking functionalities were installed in four steps *via* conjugation of propargylamine and acryloyl chloride through the carboxyl and unprotected amino groups of (Boc-Cys-OH)_2_, respectively (see ESI† for details on the **DFC** synthesis and characterisation).

In step 2, BA receptor units are introduced *via* (3-acrylamidophenyl)boronic acid (**AM-BA**) that is incubated for 30 min at an optimised pH (8.5) with a template target glycoprotein. Multiple boronate esters are formed reversibly between the **AM-BAs** and the carbohydrate structures of the glycoprotein template. The pre-assembled glycoprotein–**AM-BA** complex is then grafted on the **DFC** SAM *via* acrylamide co-polymerisation, affording the creation of spatially arranged sets of BAs on the surface that are specific for the target glycoprotein (step 3). Importantly, this unique multi-BA containing binding domain will offer an ideal covalent binding match to the carbohydrate fragments of the target glycoprotein. In order to provide complimentary allosteric specificity, a mould or imprint is created around the glycoprotein template at the surface by so-called *click* chemistry functionalization of the alkynes of the **DFC** on the SAM by reacting azide-terminated heptaethylene glycol (**Az-OEG**) moieties with the terminal alkynes on the **DFC** SAM *via* a copper-catalysed alkyne–azide cycloaddition (CuCAAC) reaction (step 4). Apart from building a molecular scaffold around the template, the OEG moieties prevent non-specific protein interaction on the surfaces (Table S5†) and provide hydrophilicity and hydrogen bonding binding sites within the imprinted surface nanocavities. The glycoprotein targets are removed by washing under acidic conditions (step 5). The hierarchical molecular construction of the glycoprotein recognition platform – a critical component of our approach – enables control over the shape, size and covalent recognition sites of the resulting cavity with a level of control that could not be achieved by any other technique. As a result, the generated sensor has greater analyte selectivity and problems such as non-specific binding and entrapment of the template are mitigated.

Surface characterisation at each step of fabrication was performed by contact angle, ellipsometry and X-ray photoelectron spectroscopy (XPS), see ESI† for full details. High-resolution XPS spectra of S 2p, N 1s and C 2s unambiguously demonstrate the presence of the **DFC** SAM on the gold surface (Fig. S18†). The XPS analysis (Fig. S20 and S22†) has established that both **AM-BA** and **Az-OEG** can be incorporated on the **DFC** SAMs with near-quantitative grafting efficiencies, providing the required molecular control over the position and density of both **AM-BA** and **Az-OEG** molecules on the **DFC** SAM.

**Fig. 2:**
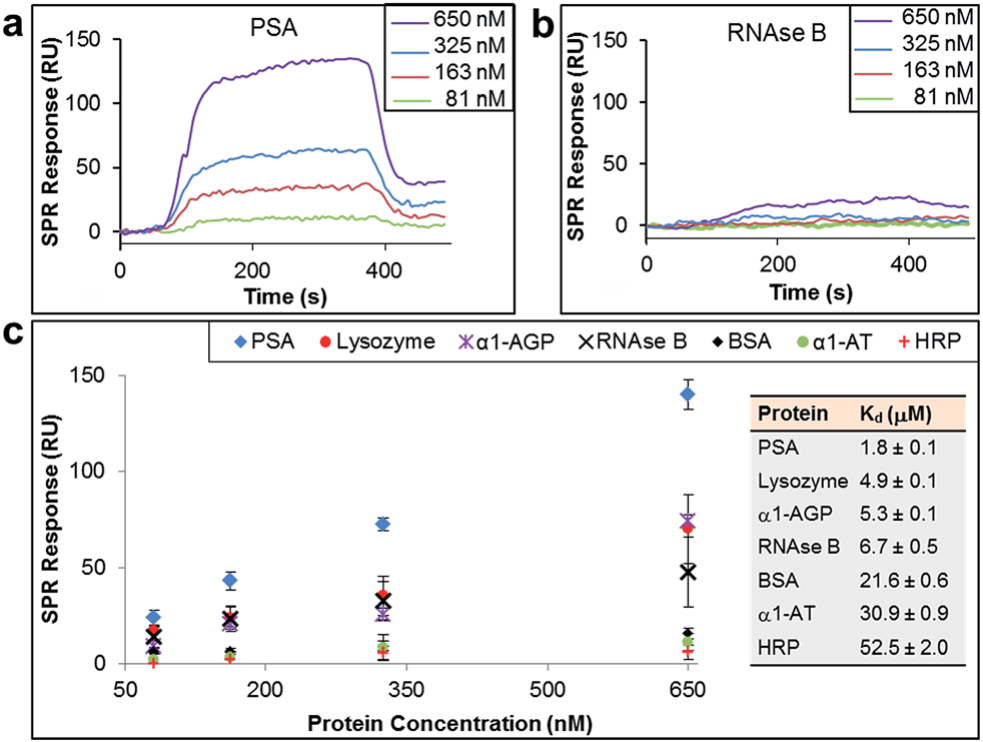
SPR sensorgram traces performed with PSA-imprinted surfaces on the SPR chip and different concentrations of (a) PSA and (b) RNAse B flowed over the surface. (c) SPR responses at equilibrium against the concentration of injected protein, PSA, lysozyme, α-1-acid glycoprotein (α1-AGP), RNAse B, bovine serum albumin (BSA), α-1-antitrypsin (α1-AT) and horseradish peroxidase (HRP), from which dissociation constants (*K*_d_) have been obtained.

The binding affinity and selectivity of the protein-imprinted surfaces for chosen targets was investigated by surface plasmon resonance (SPR) spectroscopy. Initially, we selected the glycoprotein prostate specific antigen (PSA) as the template (Fig. 2a–c). PSA is a biomarker for prostate cancer, and currently there is the urgency for its accurate detection and hence reliable diseases diagnosis. Sensing surfaces, prepared with PSA as template, showed high binding affinity with a dissociation constant (*K*_d_) of 1.8 μM, a value comparable to those of antibodies specific for PSA (typically with values in the nM to μM range).^22,23^ Furthermore, SPR analysis permitted detection of PSA at nM levels with excellent reproducibility. Surface coverage for PSA was found to range between 0.024 ng mm^–2^ and 0.140 ng mm^–2^ (100 response units (RUs) ∼ 0.1 ng mm^–2^ (ref. 24 and 25)), depending on the concentration of PSA to which the MI surface was exposed to. Calculations on surface coverage, as described in the ESI,† established that the imprinted surfaces can attain high surface coverage (70%) by PSA, with the remaining OEG non-nanocavity areas on the surface providing the desired interprotein distance for efficient binding affinity and selectivity.

In order to establish the selectivity scope of our new sensing platform, we surveyed a panel of glycosylated and non-glycosylated proteins that vary in, amongst other properties, molecular dimensions, degree of glycosylation and isoelectric point (Fig. 2c and Table 1). The PSA-imprinted surface exhibited excellent selectivity towards PSA, with all other proteins showing a significantly reduced affinity (Fig. 2c). The PSA-imprinted surface revealed a 3–30 fold selectivity to PSA over other glycosylated and non-glycosylated proteins. The difference in the magnitude of the binding affinity between the non-targeted proteins appears to be primarily attributed to their molecular size (Table 1), an allosteric effect, in which proteins of similar or smaller size to that of the target PSA displayed lower binding affinities than PSA but higher binding affinities than the other larger proteins examined. There is no observable general trend in the amount of non-target protein bound to the imprinted surface with isoelectric point. Note, however, that positively charged proteins at pH 8.5 are more prone to interact with the negatively charged boronate ion species present in the imprinted surfaces. Thus, it is reasonable to explain the higher affinity of lysozyme among the non-target proteins for the PSA-imprinted surfaces based on Coulombic interactions.

**Table 1:** Molecular dimensions, degree of glycosylation and isoelectric point of the different proteins

Protein	PSA	Lysozyme	α1-AGP	RNAse B	BSA	α1-AT	HRP
Molecular dimensions (nm × nm × nm)	4.4 × 4.1 × 5.1^*a*^	2.8 × 3.2 × 3 (ref. 26)	5.9 × 4.2 × 3.9^*a*^	3.8 × 2.8 × 2.2 (ref. 27)	14 × 4 × 4 (ref. 28)	7 × 3 × 3 (ref. 29)	4.0 × 6.7 × 11.7 (ref. 30)
Degree of glycosylation (%)	8.3	0	45	9	0	5	21
Isoelectric point	6.2–7.5 (ref. 31)	11.1 (ref. 32)	2.8–3.8 (ref. 33)	9.2–9.6 (ref. 34)	4.7 (ref. 35)	4.5–5.5 (ref. 36)	9 (ref. 37)

^*a*^Protein molecular dimensions were estimated using ChemBio Ultra 3D as described in the ESI.

If BA groups in the nanocavities were interacting non-selectively with saccharide fragments of glycoprotein analyte, one would expect a higher degree of glycosylation in non-target proteins would lead to higher binding affinities, *i.e.* more saccharides equals more potential boronic ester formation. That this is not the case, and no such trend is observed, provides convincing evidence that our covalent binding arrays of BAs within our nanocavities are indeed positioned ideally for multipoint, and thus stronger, binding to the target against which the surface imprint was made. Indeed, it is remarkably that ribonuclease (RNAse) B, which is a smaller glycoprotein than PSA with similar degree of glycosylation, produced a very low SPR response when evaluated at concentrations as high as 650 nM (Fig. 2b), thus providing even more evidence that our new nanocavity sensor is target selective. It is important to note that OEG-terminated surfaces created without the glycoprotein–**AM-BA** complex, *i.e.* a surface devoid of BA-containing nanocavities, displayed minimal non-specific protein binding, with SPR responses below 20 response units (Table S5†). Thus, the low binding of RNAse B to the PSA-imprinted surface provide evidence that we have created spatially arranged sets of BAs on the surface that are specific for the target PSA glycoprotein.

**Fig. 3:**
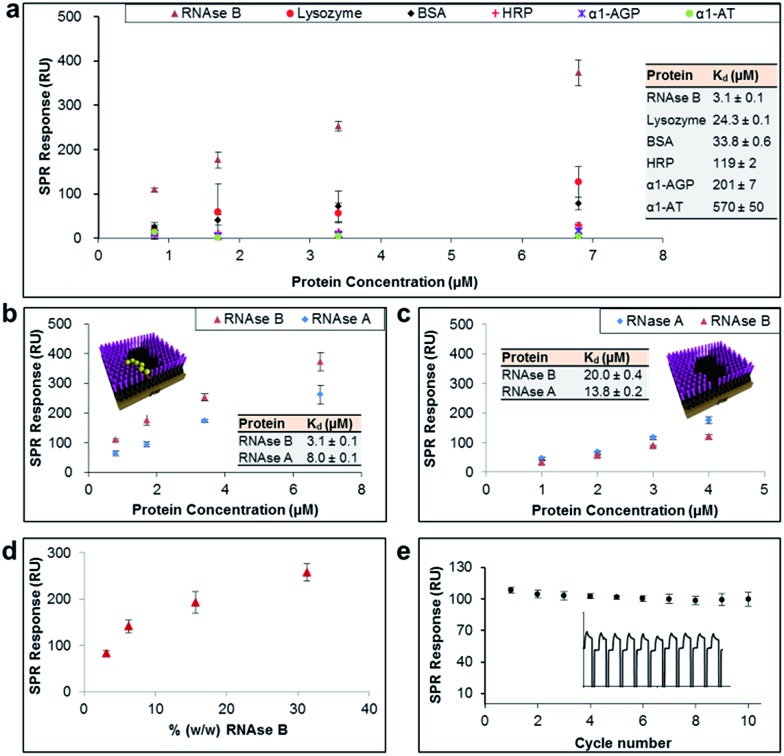
(a) SPR responses at equilibrium obtained from RNAse B-imprinted surfaces against the concentration of injected protein, from which dissociation constants (*K*_d_) have been obtained. (b and c) SPR responses at equilibrium obtained from RNAse B-imprinted surfaces, which were prepared in the (b) presence and (c) absence of BA carbohydrate receptors, against the concentration of injected RNAse B and A proteins. (d) SPR responses at equilibrium obtained from pre-conditioned RNAse B-imprinted surfaces against the % (w/w) of RNAse B in 0.5% serum solution. (e) SPR responses at equilibrium from 10 SPR cycles (as shown in the inset), which were performed using the RNAse B-imprinted surface.

The general applicability of our sensor construction strategy was evaluated using another template, RNAse B. As shown in Fig. 3a, RNAse B-imprinted surfaces exhibited greater affinity towards the templated analyte. As for the PSA-imprinted surfaces, the RNAse B-imprinted surfaces also provide a high surface coverage (77%) by RNAse B, highlighting the consistency of the experimental design.

RNAse B-imprinted surfaces revealed excellent specificity towards the templated analyte, displaying 10–200 fold selectivity to RNase B over other glycosylated and non-glycosylated proteins. Although RNAse B and lysozyme have not so dissimilar dimensions and isoelectric points (Table 1), the RNAse B-imprinted surface revealed a 8-fold enhanced selectivity for RNAse B over lysozyme, supporting the notion that pre-templated, and thus highly organised and spatially arranged, BAs within the nanocavity of the imprinted binding pocket dramatically contribute to the selectivity and affinity of the imprinted surface to the target analyte.

To investigate further the importance of the BA fragments in selectivity of the prepared sensor surfaces, RNAse B-imprinted surfaces were prepared in the presence and absence of **AM-BA** and their affinity and selectivity towards RNAse B and its non-glysosylated counterpart, RNase A, assessed (Fig. 3b and 3c). The BA-containing RNAse B-imprinted surface bound more RNAse B than RNase A, indicating that the stronger interactions are dictated by the presence of the glycan on RNAse B, and in turn its specific covalent bond formation with the spatially immobilised BA moieties within the nanocavities. The weaker RNAse A interactions are considered to be arisen to some extent from Coulombic interactions between the known^38^ positively charged RNAse A domain along its longest axis and the negatively charged boronate ion species present in the imprinted surfaces. Bare RNAse B-imprinted surfaces (absence of BA molecules in the nanocavities) exhibited lower affinity and rather poor specificity, capturing the glycoprotein to which it was templated and its non-glycosylated counterpart in a similar fashion. The bare RNAse B-imprinted surfaces resulted in about 7-fold reduced affinity to RNAse B compared with the BA-containing RNAse B-imprinted surface. These observations further highlight that the overall binding strength and selectivity of the imprinted surface towards the target glycoprotein arises from two distinct effects: shape or allosteric matching and specific covalent interactions between the boronate ion and the saccharide residues of the glycoprotein.

In order to demonstrate the *real life* utility of our sensor constructs we investigated the sensitivity of the imprinted surfaces for the target glycoprotein in complex biological conditions such as in serum. Serum is a complex biological media which comprises a wide array of proteins found in blood, with the exception of those involved with clotting. This includes proteins such as, but not limited to, albumin, immunoglobulin, haemoglobin and globulin. Furthermore, serum also contains a number of other compounds including lipids, steroid and peptide hormones. Simultaneous adsorption of RNAse B (ranging from 0.01 mg ml^–1^ to 0.1 mg ml^–1^) and 0.5% serum (*i.e.* 0.32 mg ml^–1^) on RNAse B-imprinted surfaces was monitored by SPR. In order to eliminate the background signal, the RNAse-B imprinted surfaces were initially pre-conditioned with 0.5% serum, thereby allowing it to bind to all potential sites of non-specific interaction. The pre-conditioned RNAse-B imprinted surfaces were shown to provide highly sensitive detection for RNAse B at levels as low as 3% (w/w) (Fig. 3d). The slightly reduced affinity of RNAse B towards the pre-conditioned RNAse-B imprinted surfaces (*K*_d_ = 6.5 μM ± 0.2) in comparison to the non-preconditioned RNAse-B imprinted surfaces (*K*_d_ = 3.1 μM ± 0.1) can be explained as a result of the elimination of the non-specific contribution to the overall binding affinity of RNAse B to the imprinted surface and/or serum competition for binding sites. The imprinted surfaces were also shown to be remarkably stable for more than 10 cycles of binding and regeneration of the surface (Fig. 3e).

## Conclusions

The hierarchical bottom-up assembly strategy can provide a universal route for the rational design and fabrication of glycoprotein sensors on surfaces, and thus amenable to device fabrication, with precise and predictable structures for glycoprotein recognition. The specificity with which our functional nanocavity sensor platform interacts with its target and its robustness, combined with the fact that it can be easily produced for target glycoproteins, gives this platform great potential for incorporation into biosensors^39^ and protein separation devices^40^ with applications in many areas such as biomedical diagnostics, pharmaceutical industry, defence and environmental monitoring.

## Experimental

### Surface preparation

#### Self-assembled monolayer (SAM) preparation

Polycrystalline gold substrates were purchased from George Albert PVD (Germany), and consisted of a 50 nm gold layer deposited onto glass covered with a thin layer of chromium. The Au substrates were cleaned by immersion in piranha solution (7 : 3, H_2_SO_4_ : H_2_O_2_) at room temperature for 10 min (**caution**: piranha solution reacts violently with organic compounds and should be handled with care). Samples removed from the piranha solution were immediately rinsed with Ultra High Pure (UHP) water, followed by rinsing in HPLC grade methanol (Fischer Scientific, UK) each for 1 min. Immediately after cleaning, the substrates were immersed in freshly prepared 0.1 mM methanolic solutions of **DFC**. Contact angle kinetics studies demonstrated that SAMs were fully formed after 18 hours immersion.

#### Fabrication of molecularly imprinted surfaces

SAMs of **DFC** were formed as described above. A solution of **AM-BA** (20 μl of a 7.5 mM solution) was mixed with 20 μl solution of template protein (20 μl of a 250 μM solution) in phosphate buffer solution (2 ml PBS at pH 8.5), and incubated for 30 minutes to permit the formation of **AM-BA**: protein complexes. To the solution thus obtained the **DFC** SAMs were placed. To this, a solution of ammonium persulfate (100 ml of a 175 mM solution) and TEMED (1 μl) was added to trigger the crosslinking between the **DFC** SAMs and the **AM-BA**: protein complex for 15 minutes. To this solution, **Az-OEG** (1 μl of a 5 mM aqueous solution) was added. After 10 minutes, the Cu-AACA reaction was initiated by the addition of a solution of pre-prepared catalyst (copper sulfate (15 ml of a 40 mM solution) and sodium ascorbate (15 ml of a 100 mM solution)). The mixture was allowed to react for 4 hours, after which time, the modified gold substrates were rinsed liberally with UHQ water for 3 min to remove the bound template protein.

#### Surface plasmon resonance analysis (SPR)

SPR experiments were performed with a Reichert SR7000DC Dual Channel Spectrometer (Buffalo, NY, USA) at 25 °C. Modified gold-coated SPR chips were deposited on the base of the prism using index-matching oil. Prior to the binding studies, a baseline was established by running degassed PBS (pH 8.5) through the SPR at a flow rate of 25 μl min^–1^. The modified gold surfaces were subsequently exposed to protein solutions in PBS injected at 25 μl min^–1^ for 5 min, after which a ten min dissociation phase was introduced by flowing buffer over the surface. Data sets were processed and analyzed using Scrubber 2 (BioLogic Software, Campbell, Australia). The SPR responses at equilibrium (*R*_eq_) were plotted against the concentration of injected protein (*C*_p_) and fitted to a 1 : 1 steady-state affinity model. The model utilises a nonlinear least-squares regression method to fit data to the Langmuir adsorption isotherm (eqn (1)). *K*_d_ is the dissociation constant for binding of the proteins to the MI surfaces and *R*_max_ is the maximum response if all available MI binding sites are occupied.1
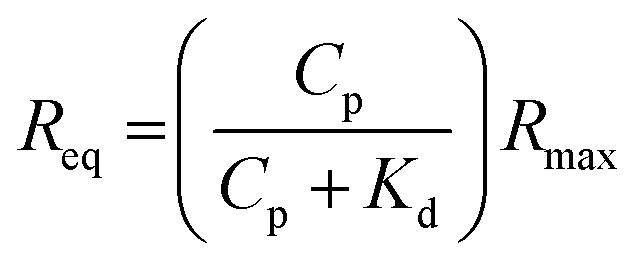



For the serum experiments, the blocking was performed by running degassed PBS containing 0.5% serum through the SPR at a flow rate of 25 μl min^–1^ for 30 min. The modified gold surfaces were subsequently exposed to RNAse B (0.85 μM, 1.7 μM, 3.4 μM or 6.8 μM) and 0.5% serum solutions as described above. The acidic regeneration solution, which was adjusted to pH 5.0, comprised equal volumes of oxalic acid, phosphoric acid, formic acid and malonic acid, each at 0.075 M.

#### Estimation of PSA and α1-AGP protein molecular dimensions

Protein molecular dimensions were estimated using ChemBio Ultra 3D (Cambridgesoft, Perkin Elemer, USA) from protein crystal structures downloaded from RCSB (Research Collaboratory for Structural Bioinformatics) Protein data bank (http://www.rcsb.org/).
