# Supplementary material for: Selective glycoprotein detection through covalent templating and allosteric click-imprinting
Source: Chem Sci. 2015 Jun 17;6(9):5114–9. doi: 10.1039/c5sc02031j (PMC5666680; doi:10.1039/c5sc02031j)
Supplement: Supplementary file 1 [file SC-006-C5SC02031J-s001.pdf]

Electronic Supplementary Material (ESI) for Chemical Science.
This journal is © The Royal Society of Chemistry 2015

Selective glycoprotein detection through covalent templating and allosteric *click*-imprinting

Alexander Stephenson-Brown,[†] Aaron L. Acton,[†] Jon A. Preece,[§] John S. Fossey,[§,*] Paula M. Mendes[†,*]

[†]*School of Chemical Engineering and* [§]*School of Chemistry, University of Birmingham, Birmingham, B15 2TT, UK*

SUPPORTING INFORMATION

Contents

1. Chemicals and Materials 3
2. Synthetic procedures 3
 2.1 Chromatography. 3
 2.2 NMR Spectroscopy. 4
 2.3 Mass Spectrometry. 4
 2.4 Infrared Spectroscopy (IR). 4
 2.5 Melting Points. 4
 2.6 Thin-Layer Chromatography (TLC). 4
3. Synthesis of DFC 5
  3.1 (2*R*,2'*R*)-Bis(2,5-dioxopyrrolidin-1-yl)3,3'-disulfanediylbis(2-((tert-butoxycarbonyl)amino)propanoate) (1). 5
  3.2 Di-tert-butyl((2*R*,2'*R*)-disulfanediylbis(1-oxo-1-(prop-2-yn-1-ylamino)propane-3,2-diyl))dicarbamate (2). 5
  3.3 (2*R*,2'*R*)-3,3'-Disulfanediylbis(2-amino-*N*-(prop-2-yn-1-yl)propanamide) (3). 6
  3.4 *N*,*N*'-((2*R*,2'*R*)-Disulfanediylbis(1-oxo-1-(prop-2-yn-1-ylamino)propane-3,2-diyl))diacrylamide (DFC). 6
4. NMR Spectra 8
5. Surface Modification and Characterisation 16
 5.1 Crosslinking between the DFC SAM and AM-BA 16
 5.2 O-(2-Azidoethyl)heptaethylene glycol (Az-OEG) immobilisation on the DFC SAM via a copper catalysed azide alkyne cycloaddition (Cu-AACA) 16

5.3 Characterization of SAMs and molecularly imprinted surfaces 16
5.3.1 Contact Angle. 16
5.3.2 Ellipsometry. 16
5.3.3 X-Ray photoelectron spectroscopy (XPS). 17
5.4 Contact angle and ellipsometry analysis of the DFC SAM and grafted AM-BA and Az-OEG on the DFC SAM 17
5.5 XPS analysis of the DFC SAM and grafted AM-BA and Az-OEG on the DFC SAM 18
6. SPR analysis of binding of proteins on DFC SAMs grafted with Az-OEG 23
7. References 24

1. Chemicals and Materials

All chemicals, reagents and proteins were purchased from Sigma Aldrich (UK) with the following exceptions: HPLC grade methanol, triethylamine (TEA) and tetrahydrofuran (THF), which were purchased from Fischer Scientific (UK). Prostate specific antigen was purchased from Antibodies Online (Germany). All reagents were used as supplied without further purification, with the exception of THF which was re-distilled onsite using PureSolv EN Solvent Purification System (Innovative Technology Inc., UK). Unless otherwise stated all water used was deionised.

2. Synthetic procedures

DFC (4) was synthesised through a multistep route as illustrated in **Scheme S1**. The carboxylic acid groups of the commercially available starting material *N*α,*N*α′-di-Boc-L-cystine were activated with dicyclohexylcarbodiimide (DCC) and coupled with *N*-hydroxysuccinimide (NHS) over 18 hours at room temperature, to produce the NHS ester **1**. Compound **1** was then reacted with propargylamine over 4 hours at room temperature to produce **2**. Deprotection of the boc protected amines in **2** was achieved using trifluoroacetic acid over 24 hours at room temperature to produce **3**. Acrylic groups were then coupled to the free amines of **3** via reaction with acryloyl chloride over 4 hours to obtain the di-functional cystine, **DFC**.

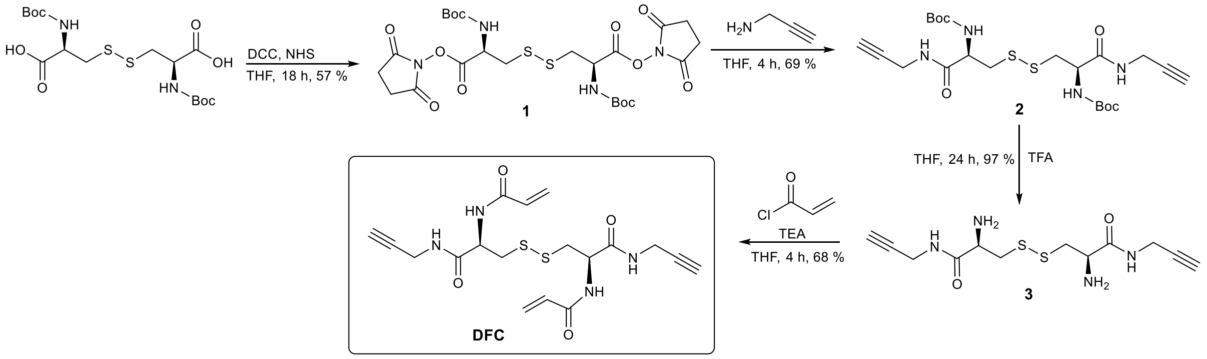

Scheme S 1 Synthesis of **DFC**.

2.1 Chromatography. Flash chromatography was performed on a Teledyne Isco CombiFlash Rf 200 using RediSep Rf silica flash columns. Ethyl acetate and hexane where used as solvents. Detection of eluted compounds was achieved using UV and evaporative light scattering detectors (ELSD).

2.2 NMR Spectroscopy. ^{1}H and ^{13}C NMR spectra were recorded on a Bruker AV300 (at 300MHz and 75MHz respectively) or a Bruker AVIII400 (at 400 MHz and 101 MHz respectively) at room temperature. All ^{13}C NMR spectra were recorded using the PENDANT pulse sequence. Where necessary, COSY, HSQC and NOSEY experiments were carried out to allow unequivocal assignment of signals. Chemical shifts are expressed in parts per million (ppm) down field from tetramethylsilane or relative to residual NMR solvent peak. Data was processed on MestReNova LITE v.5.2 (Mestrelab Research) and Topspin 2.0 (Bruker). The multiplicity of signals is expressed as follows: s = singlet, d = doublet, t = triplet q = quartet, m = multiplet. Coupling constants (*J*) are reported in Hz.

2.3 Mass Spectrometry. All samples were analysed by means of the Synapt G2-S HDMS system (Waters, Manchester, UK). All experimental data were acquired with a resolution of 20000. Samples were introduced into the mass spectrometer *via* the nanoAcquity system (Waters, Manchester, UK). Electrospray ionisation was performed with a capillary voltage of 3.2 kilovolts, and the sample cone was set at 40 volts.

2.4 Infrared Spectroscopy (IR). IR spectra were recorded using a PerkinElmer Spectrum 100 FTIR Spectrometer, using a universal ATR sampler (PerkinElmer). Frequencies (in wavenumbers) are listed, with the relative strength and a brief assignment of what type of bond is resonating listed in parentheses. Peaks are listed in descending numerical order. Strengths: s = strong, m = medium, w = weak, br = broad.

2.5 Melting Points. Melting points (mp) were recorded using a Stuart SMP10, using closed ended melting point tubes. Values stated are uncorrected.

2.6 Thin-Layer Chromatography (TLC). TLC was carried out on aluminium plates coated with silica gel 60 F254 (Merck 5554). The TLC plates were visualised using either potassium manganate or ninhydrin dip and dried with a heat gun.

3. Synthesis of DFC

3.1 (2*R*,2'*R*)-Bis(2,5-dioxopyrrolidin-1-yl)3,3'-disulfanediylbis(2-((tert-butoxycarbonyl)amino)propanoate) (1).

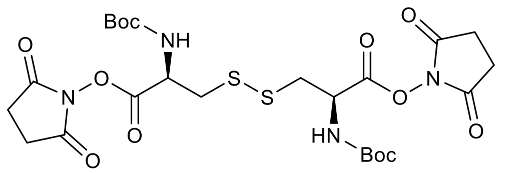

To a solution of Di-Boc-L-cystine (2.161 g, 5.14 mmol) in dry THF (50 mL) was added, *N*-hydroxysuccinimide (1.355 g, 11.3 mmol) and dicyclohexylcarbodiimide (2.327 g, 11.3 mmol). The solution was stirred at room temperature overnight and the resulting precipitate was removed by filtration and the solvent removed *in vacuo.* The crude residue was dissolved in CH_2Cl_2 (20 mL), washed with sat. $NaHCO_{3(aq)}$ (3 x 25 mL) and the aqueous phases extracted further with CH_2Cl_2 (3 x 25 mL). The organic extracts were combined, dried over $MgSO_4$, filtered and the solvent removed *in vacuo* to yield **1** (1.857 g, 57 %) as a colourless crystalline solid. FTIR (cm^{-1}) [3376; m, br; N-H], [1749; S; C=O]; ^1H NMR (400 MHz, $CDCl_3$) δ 5.41 (2H, d, *J* = 7.5, 2 × N*H*COO), 4.62 (2H, dd, *J* = 12.5, 5.0, 2 × NHC*H*CH_2), 3.78 (8H, s, 4 x NCOCH_2CH_2), 3.18 (4H, d, *J* = 5.0, 2 × NHCHCH_2), 1.46 (18 H, s, 6 x NHCOOCH_3); ^{13}C NMR (101 MHz, $CDCl_3$) δ 52.7 (s, SHCH_2*C*H), 52.6 (s, NCO*C*H_2CH_2), 41.3 (s, SH*C*H_2CH), 28.3 (s, NHCOO*C*H_3); Mass spec: calculated: 657.2 $[M+Na]^+$, observed: 657.2 $[M+Na]^+$ $C_{24}H_{34}N_4O_{12}S_2$; mp: 97-99 °C.

3.2 Di-tert-butyl((2*R*,2'*R*)-disulfanediylbis(1-oxo-1-(prop-2-yn-1-ylamino)propane-3,2-diyl))dicarbamate (2).

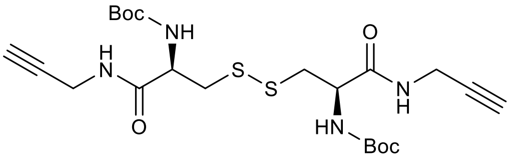

To a solution of **1** (0.420 g, 0.66 mmol) in THF (50 mL), was added propaglyamine (0.091 g, 1.65 mmol) at 0 °C. The solution was allowed to warm to room temperature and was stirred for four hours. The resulting precipitate was removed by filtration and the solvent removed *in vacuo.* The crude residue was dissolved in CH_2Cl_2 (20 mL), washed with sat. $NaHCO_{3(aq)}$ (3 x 25 mL) and the aqueous phases extracted further with CH_2Cl_2 (3 x 25 mL). The organic extracts were combined, dried over $MgSO_4$, filtered and the solvent removed *in vacuo*. The crude product was purified further by automated flash chromatography, using a silica column and an EtOAc/hexane gradient to yield **2** (0.235 g, 69 %) as a colourless solid. IR (cm^{-1}) [3290; S, br; NH], [1655; S; C=O], [1518; S; C=O]; ^1H NMR (400 MHz, $CDCl_3$) δ 8.10 (2H, t, *J* = 5.5, 2 × N*H*CH_2CCH), 5.57 (2H, d, *J* = 9.5, 2 × N*H*COO), 4.94 (2H, dtd, *J* = 11.0, 9.5, 4.0, 2 × SHCH_2C*H*), 4.09 (4H, ddd, *J* = 17.5, 5.5 and 2.5, 2 × NHCH_2CCH), 2.98 (2H, dd, *J* = 15.0, 4.0, 2 × SCH*H*CH), 2.88 (2H, dd, *J* = 15.0, 11.0, SC*H*HCH), 2.20 (2H, t, *J* = 2.5, 2 × NHCH_2CC*H*), 1.50 (18H, s, 6 × NHCOOCH_3); ^{13}C NMR (101 MHz, $CDCl_3$) δ 54.4 (s, SCH_2*C*H), 47.4 (s, S*C*H_2), 28.8 (s,

NHC*H*2CCH), 28.5 (s, NHCOOCCH_3). Mass spec: calculated 537.2 $[M+Na]^+$, observed 537.2 $[M+Na]^+$ $C_{22}H_{34}N_4O_6S_2$; HRMS: calculated 537.1817 observed 537.1827 [M+Na] $C_{22}H_{34}N_4O_6S_2$; Mp 170-171 °C.

3.3 (2*R*,2'*R*)-3,3'-Disulfanediylbis(2-amino-*N*-(prop-2-yn-1-yl)propanamide) (3).

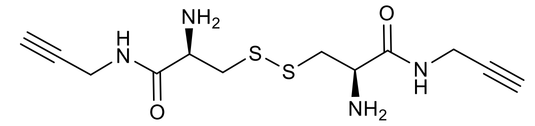

To a solution of **2** (0.410 g, 0.79 mmol) in THF (50 mL), was added TFA (1.5 mL). After stirring the solution at room temperature for 4 hours the solvent was removed *in vacuo* to yield **3** (0.246 g, 97 %), as a light brown solid IR (cm^{-1}) [3289; w, br; N-H], [1526 M/S br; NH_2], [1660; S; C=O]; ^1H NMR (400 MHz, MeOD) δ 4.24 (2H, dd, *J* = 8.5, 5.0, 2 × SCH_2CHNH_2), 4.08 (4H, d, *J* = 2.5, 2 × $NHCH_2CH$), 3.40 (2H, dd, *J* = 15.0, 5.0, 2 × SC*H*HCH), 3.10 (2H, dd, *J* = 15.0, 8.5, 2 × SCH*H*CH), 2.70 (2H, t, *J* = 2.5, $NHCH_2CCH$). ^{13}C NMR (101 MHz, MeOD) δ 78.0 (s, SCH_2CHC=O), 71.6 (s, $NHCH_2CCH$), 51.4 (s SCH_2CH), 37.3 (s, SCH_2), 33.3 (s, $NHCH_2CCH$), 28.9 (s, $NHCH_2CCH$). Mass spec: calculated 315.1 $[M+H]^+$, observed 315.1 $[M+H]^+$ $C_{18}H_{19}N_4O_2$ S_2; HRMS: observed: 315.0943 calc: 315.0949 $[M+H]^+$ $C_{18}H_{19}NaN_4O_2S_2$; Mp 79-81 °C.

3.4 *N*,*N*'-((2*R*,2'*R*)-Disulfanediylbis(1-oxo-1-(prop-2-yn-1-ylamino)propane-3,2-diyl))diacrylamide (DFC).

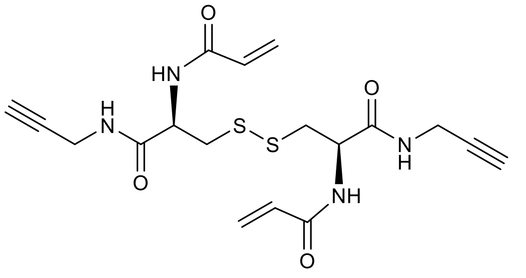

To a stirred solution of **3** (0.060 g, 0.11 mmol) and TEA (0.056 g, 0.55 mmol) in THF (25 mL) was added, acryloyl chloride (50 μL, 0.55 mmol) dropwise at 0 °C. The solution was allowed to warm slowly to room temperature and was stirred for 4 hours. The solvent was removed *in vacuo* and the crude residue was dissolved in CH_2Cl_2 (20 mL), washed with sat. $NaHCO_{3(aq)}$ (3 x 15 mL) and the aqueous phases were extracted further with CH_2Cl_2 (3 x 25 mL). The organic extracts were combined, dried over $MgSO_4$, filtered and the solvent was removed *in vacuo*. The crude product was further purified by automated column chromatography using a silica column and an EtOAc/ hexane gradient, to yield **DFC** (0.032 g, 68 %) as an off-white solid. IR (cm^{-1}) [3277; m, br; NH], [1623; S; C=O], [1647; S; C=O], [1529; S; alkene]; ^1H NMR (400 MHz, $CDCl_3$) δ 8.73 (2H, t, *J* = 5.0, 2 × $NHCH_2CH$), 6.67 (2H, d, *J* = 9.5, 2 × SCH_2CHNH), 6.44 (2H, dd, *J* = 17.0, 1.5 2 × C=OCHC*H*H), 6.21 (2H, dd, *J* = 17.0, 10.0, , 2 × C=OC*H*CH_2), 5.77 (2H, dd, *J* = 10.0, 1.5, 2 × C=OCHCH*H*), 5.61 (2H, dtd, *J* = 11.0, 9.5, 4.0, 2 × SCH_2CH), 4.16 – 4.00 (4H, m, 2 × $NHCH_2CH$), 3.08 (2H, dd, *J* = 15.0, 4.0, 2 × SC*H*HCH), 2.93 (2H, dd, *J* = 15.0, 11.0, 2 × SCH*H*CH), 2.26 (2H, t, *J* = 2.5, 2 × $NHCH_2CH$); ^{13}C NMR (101 MHz, MeOD) δ 130.0 (s, C=OCHCH_2), 126.3 (s, C=OCHCH_2), 52.4 (s, SCH_2CH), 40.6 (s, SCH_2), 28.2 (s, $NHCH_2CH$); ^1H NMR (400

MHz, MeOD) δ 6.35 (2H, dd, *J* = 17.0, 9.0, 2 × NHC=OC*H*), 6.29 (2H, dd, *J* = 17.0, 3.0, 2 × NHC=OCH*C*HH), 5.73 (2H, dd, *J* = 9.0, 3.0, 2 × NHC=OCH*C*H*H*), 4.87 (2H, dd, *J* = 9.0, 5.5, 2 × SCH_2CH), 4.01 (4H, d, *J* = 2.5 , 2 × $NHCH_2CH$), 3.21 (2H, dd, *J* = 14.0, 5.5, 2 × SC*H*HCH), 2.97 (2H, dd, *J* = 14.0, 9.0 , 2 × SCH*H*CH), 2.62 (2H, t, *J* = 2.5 2 × $NHCH_2CH$). Mass spec calculated 445.1$[M+Na]^+$, observed: 445.1 $[M+Na]^+$ $C_{18}H_{22}NaN_4O_4S_2$; HRMS: calculated: 445.0980 observed: 445.0967 [M+Na] $C_{18}H_{22}NaN_4O_4$; mp 232-235 °C.

4. NMR Spectra

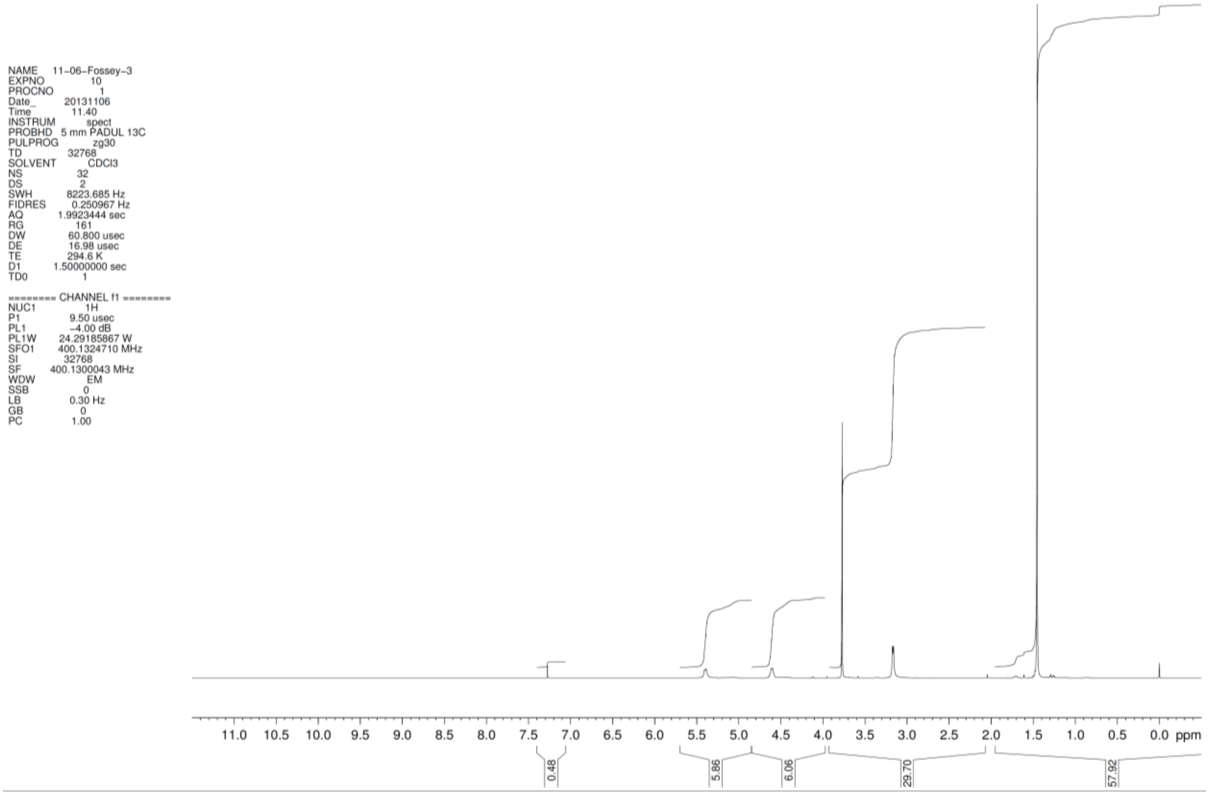

Figure S 1 ^{1}H NMR spectrum of (2R,2'R)-bis(2,5-dioxopyrrolidin-1-yl)3,3'-disulfanediylbis(2-((tert-butoxycarbonyl)amino)propanoate) (**1**).

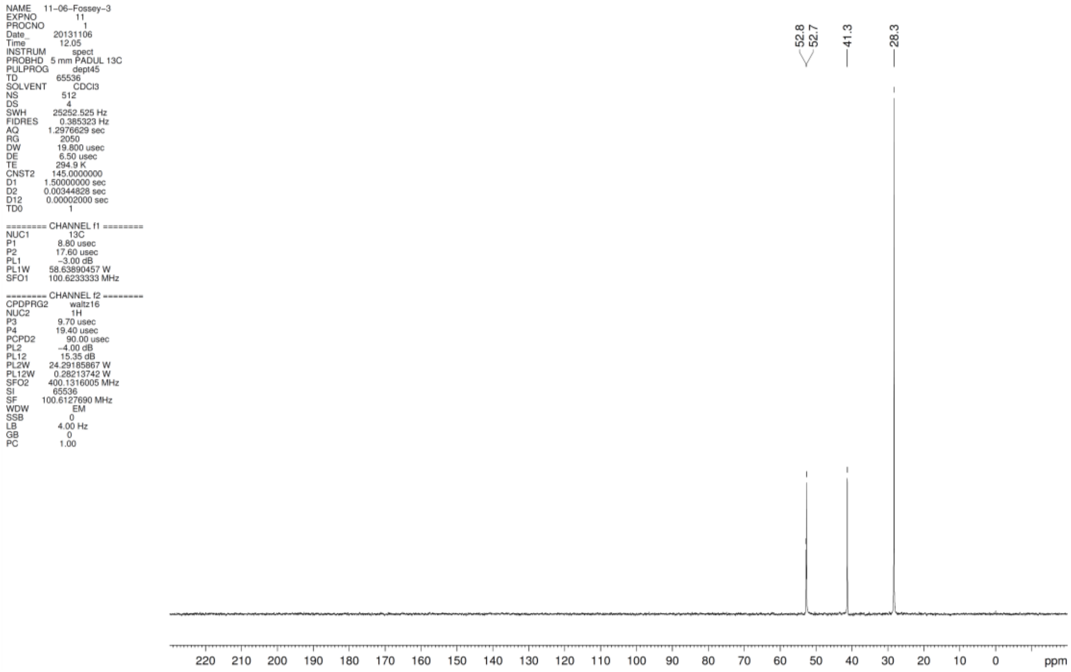

Figure S 2 ^{13}C NMR spectrum of (2R,2'R)-bis(2,5-dioxopyrrolidin-1-yl)3,3'-disulfanediylbis(2-((tert-butoxycarbonyl)amino)propanoate) (**1**).

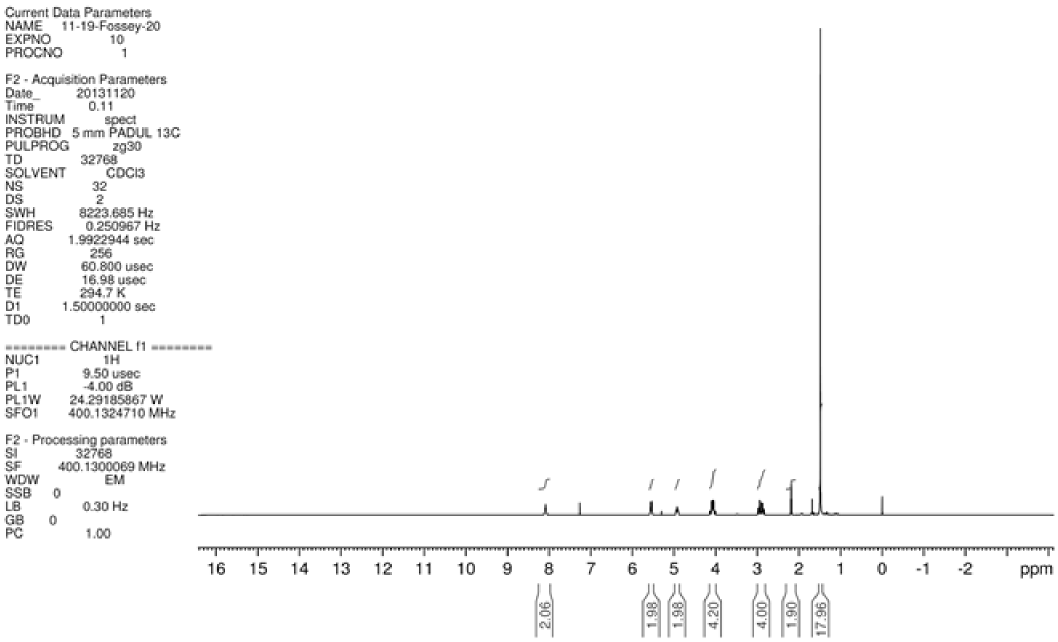

Figure S 3 ^{1}H NMR spectrum of Di-tert-butyl((2*R*,2'*R*)-disulfanediylbis(1-oxo-1-(prop-2-yn-1-ylamino)propane-3,2-diyl))dicarbamate (**2**).

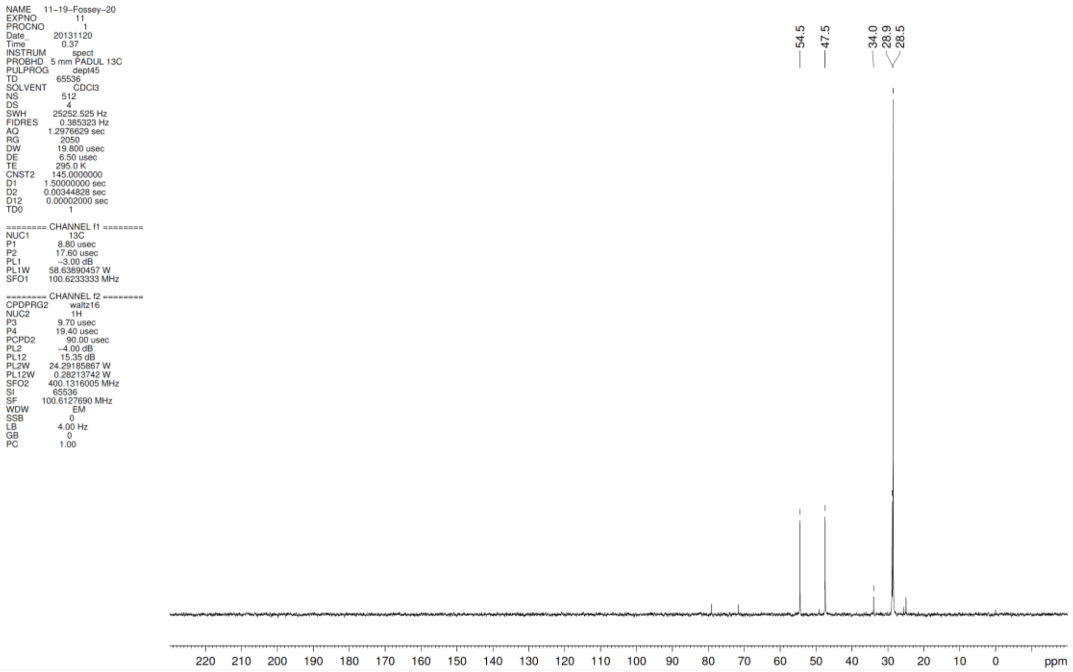

Figure S 4 ^{13}C NMR spectrum of Di-tert-butyl((2*R*,2'*R*)-disulfanediylbis(1-oxo-1-(prop-2-yn-1-ylamino)propane-3,2-diyl))dicarbamate (**2**).

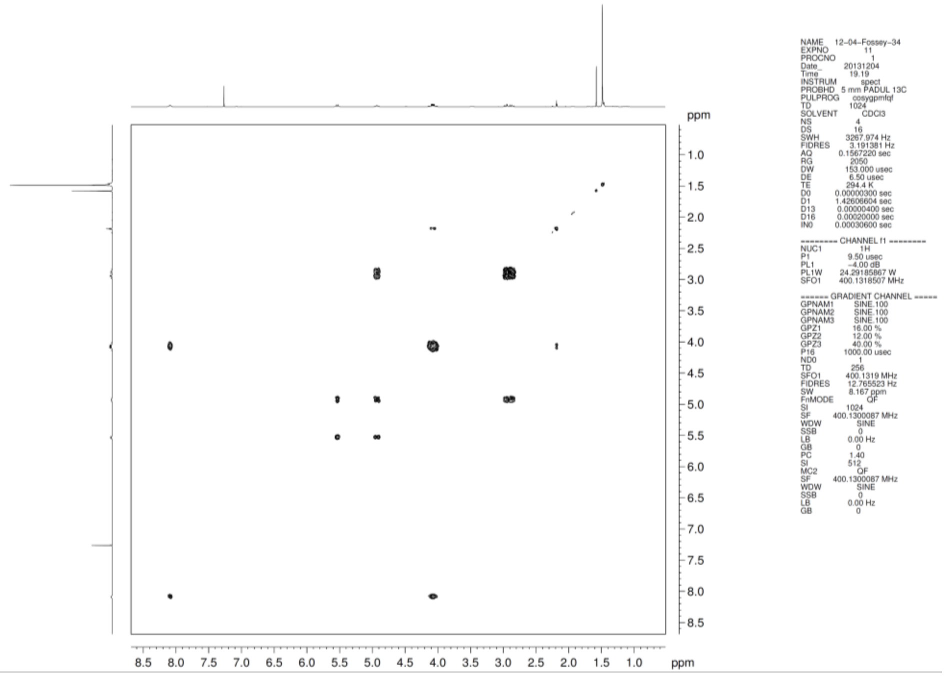

Figure S 5 COSY NMR spectra of Di-tert-butyl((2*R*,2'*R*)-disulfanediylbis(1-oxo-1-(prop-2-yn-1-ylamino)propane-3,2-diyl))dicarbamate (**2**).

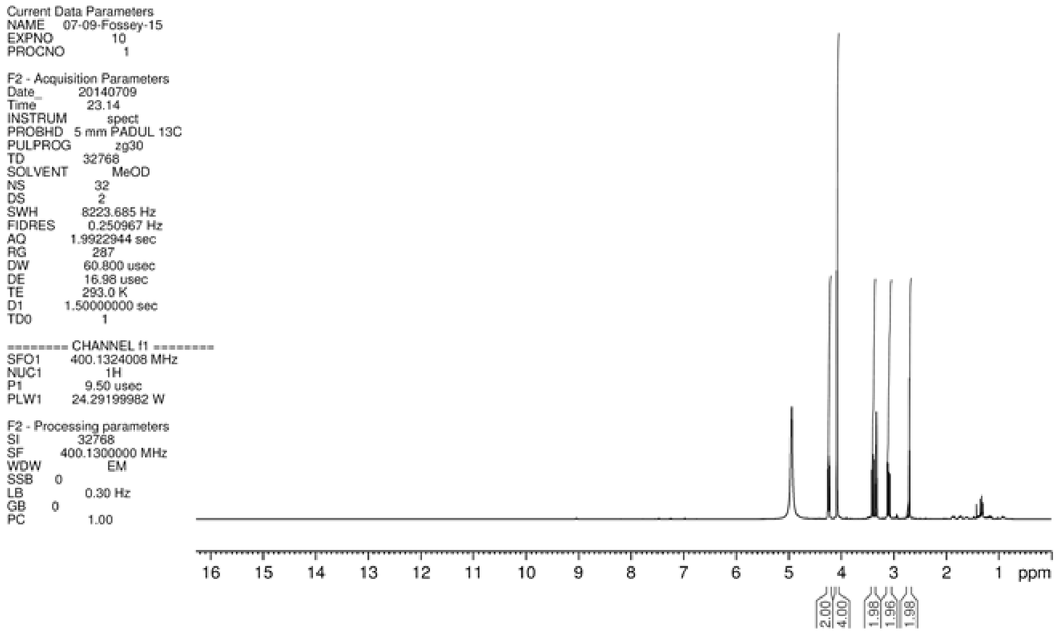

Figure S 6 ^{1}H NMR spectrum of (2*R*,2'*R*)-3,3'-disulfanediylbis(2-amino-*N*-(prop-2-yn-1-yl)propanamide) (**3**).

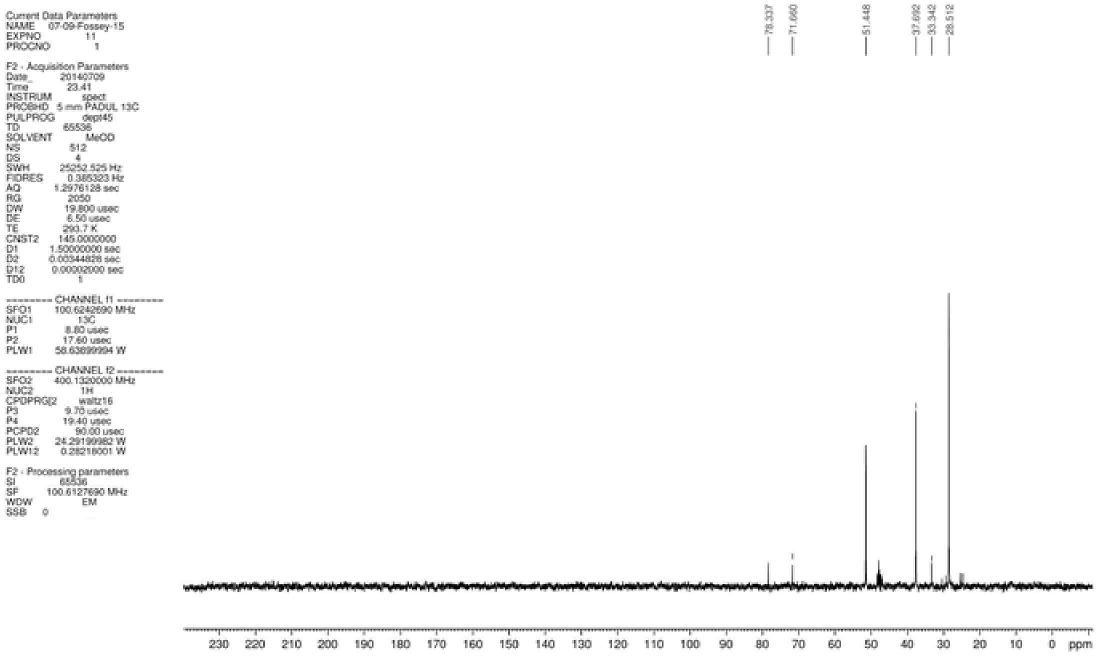

Figure S 7 ^{13}C NMR spectrum of (2*R*,2'*R*)-3,3'-disulfanediylbis(2-amino-*N*-(prop-2-yn-1-yl)propanamide) (**3**).

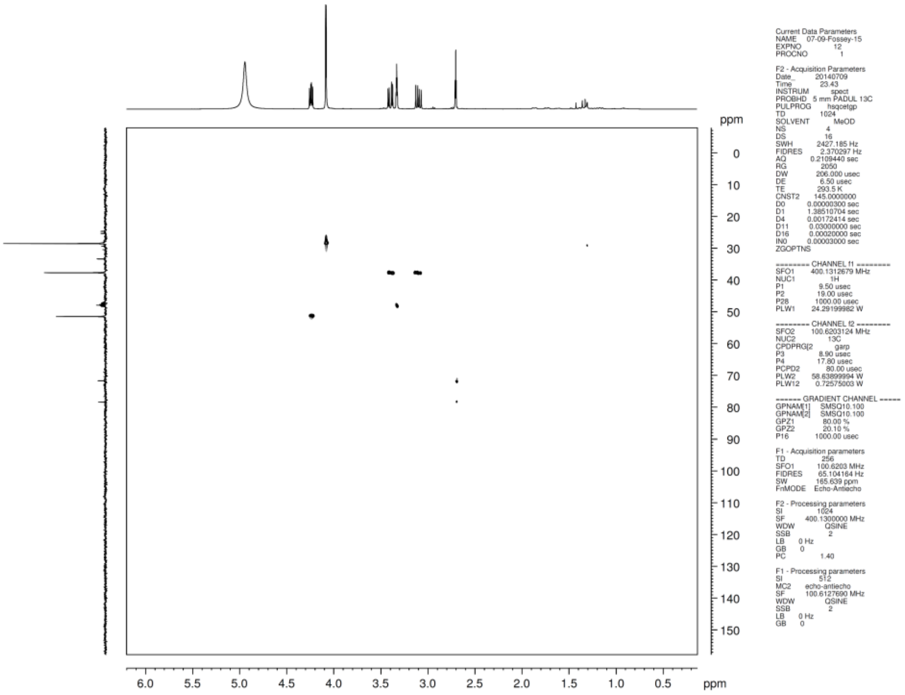

Figure S 8 HSQC NMR Spectra of (2*R*,2'*R*)-3,3'-disulfanediylbis(2-amino-*N*-(prop-2-yn-1-yl)propanamide) (**3**).

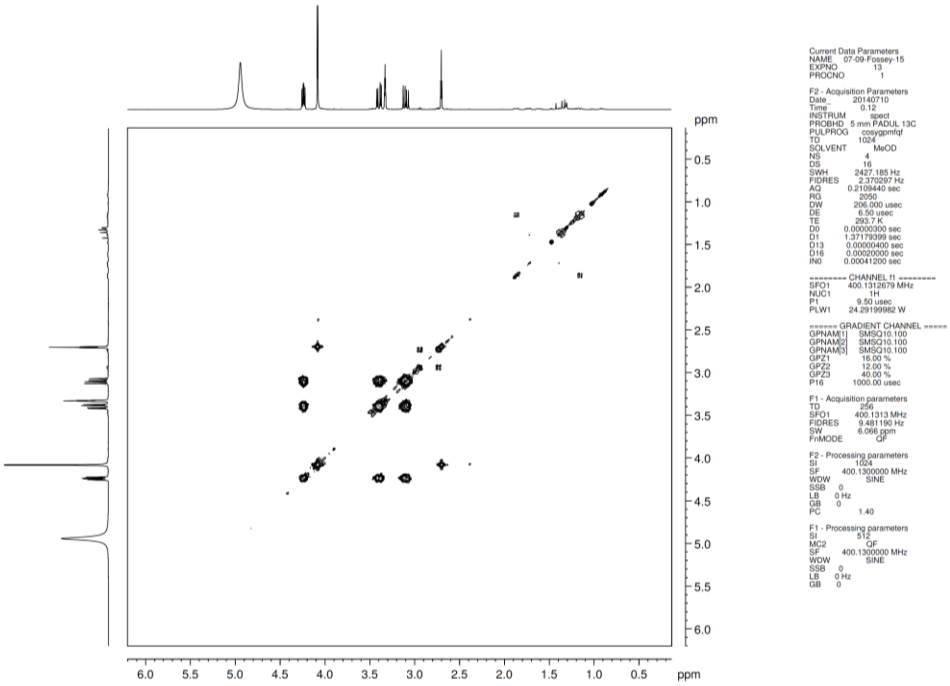

Figure S 9 COSY NMR Spectra of (2*R*,2'*R*)-3,3'-disulfanediylbis(2-amino-*N*-(prop-2-yn-1-yl)propanamide) (**3**).

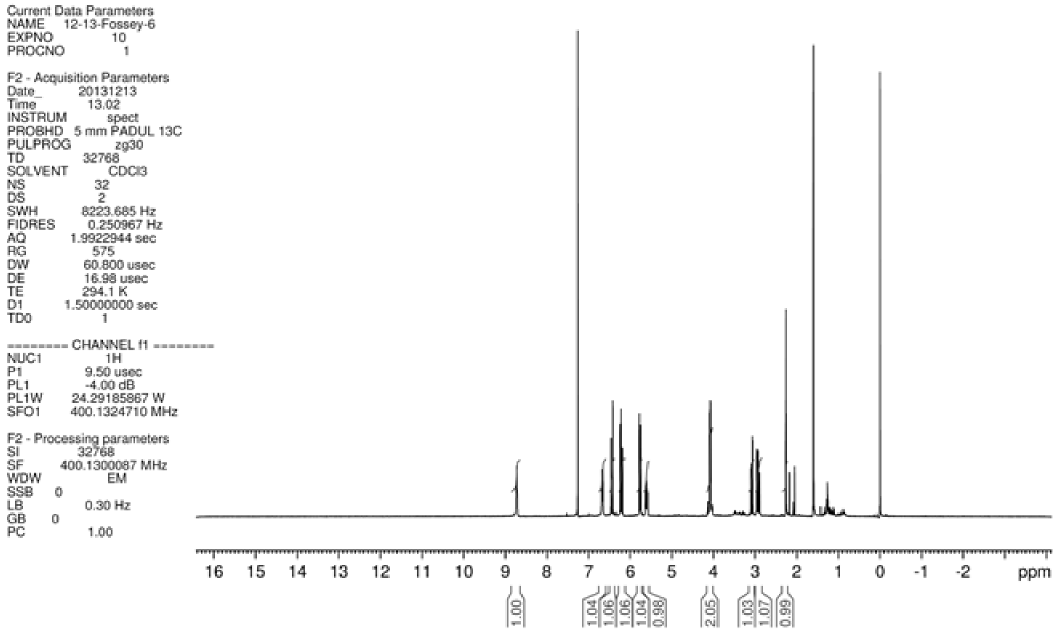

Figure S 10 ^{1}H NMR spectrum of *N*,*N*'-((2*R*,2'*R*)-disulfanediylbis(1-oxo-1-(prop-2-yn-1-ylamino)propane-3,2-diyl))diacrylamide (**DFC**).

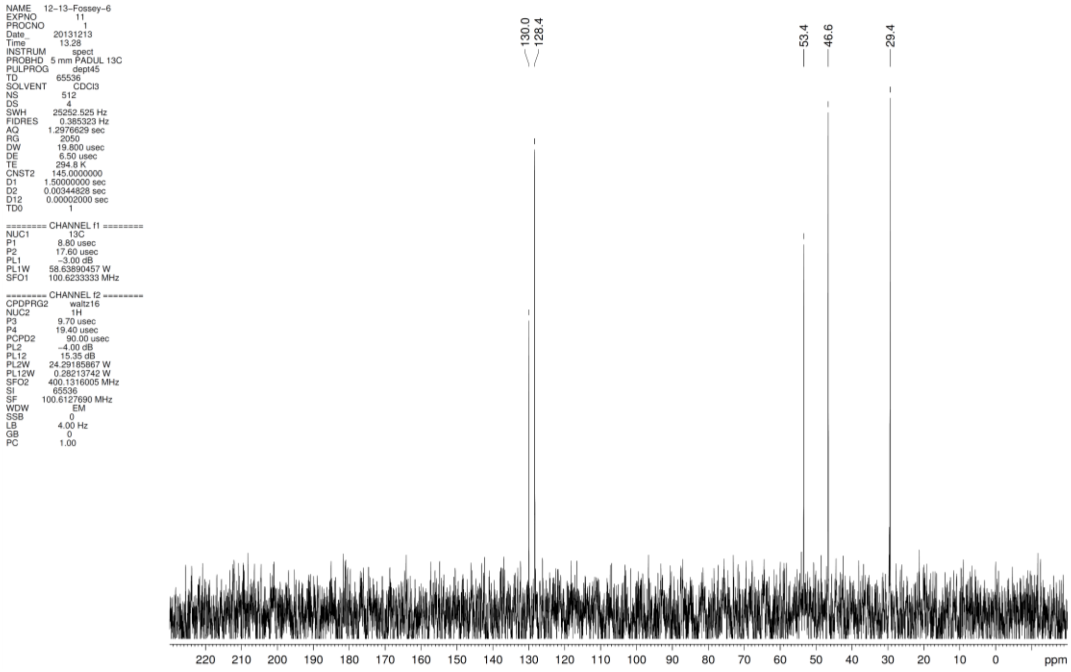

Figure S 11 ^{13}C *N*,*N*'-((2*R*,2'*R*)-disulfanediylbis(1-oxo-1-(prop-2-yn-1-ylamino)propane-3,2-diyl))diacrylamide (**DFC**).

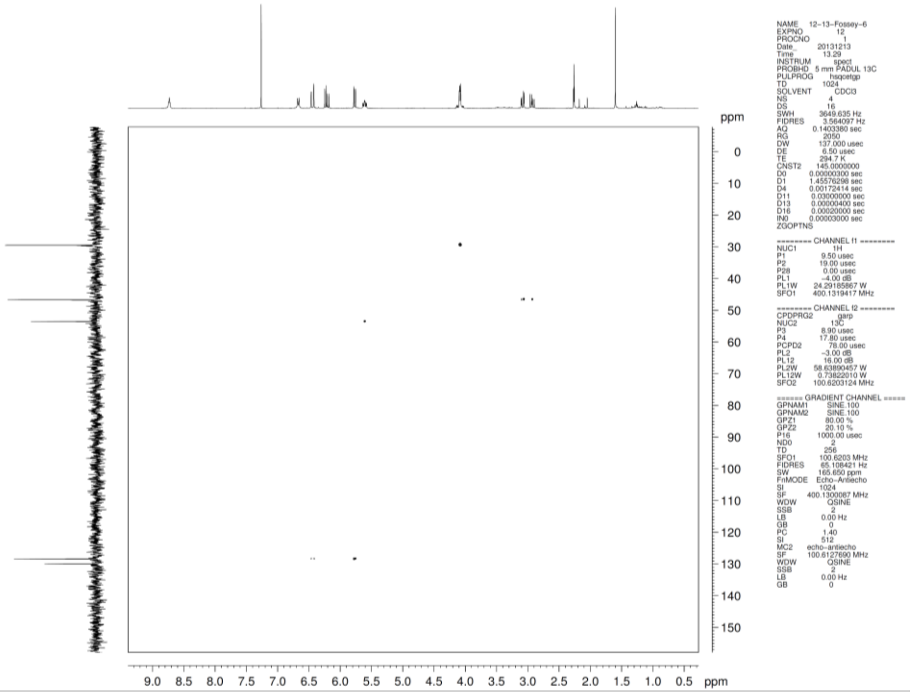

Figure S 12 HSQC spectra of *N*,*N*'-((2*R*,2'*R*)-disulfanediylbis(1-oxo-1-(prop-2-yn-1-ylamino)propane-3,2-diyl))diacrylamide (**DFC**).

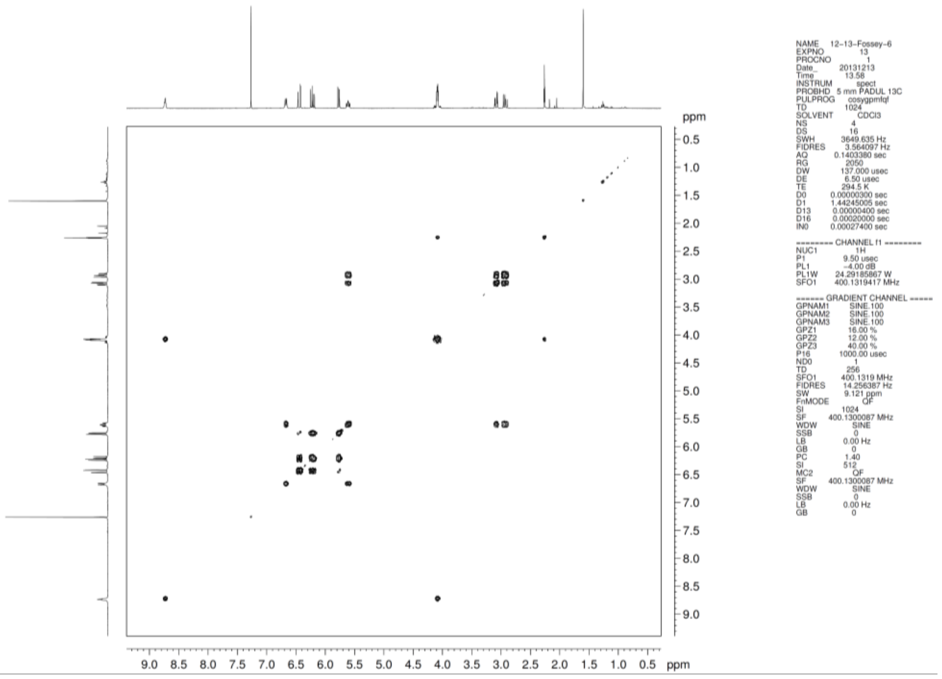

Figure S 13 COSY spectra of *N*,*N*'-((2*R*,2'*R*)-disulfanediylbis(1-oxo-1-(prop-2-yn-1-ylamino)propane-3,2-diyl))diacrylamide (**DFC**).

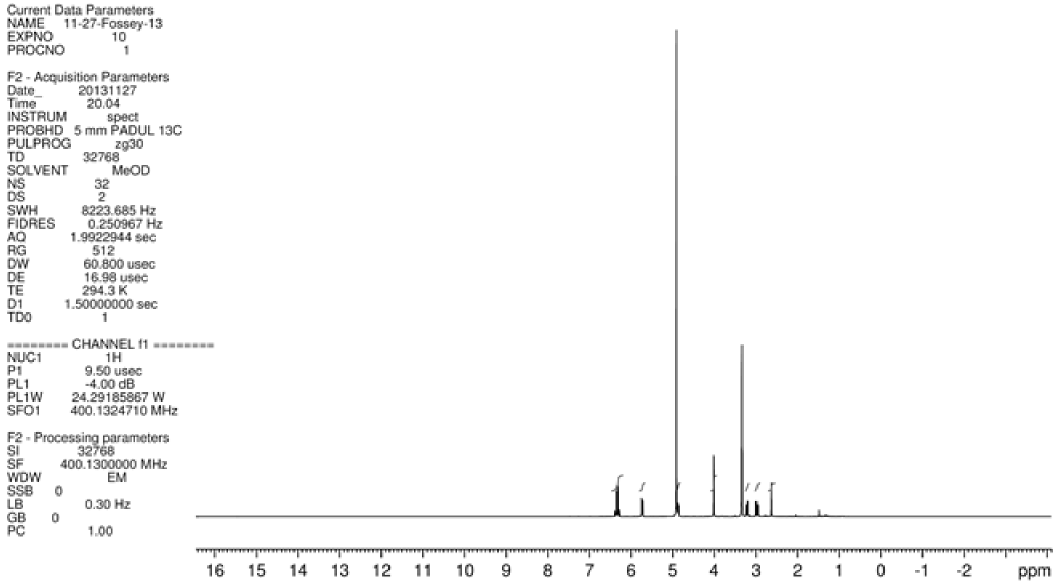

Figure S 14 ^{1}H NMR spectrum of *N*,*N*'-((2*R*,2'*R*)-disulfanediylbis(1-oxo-1-(prop-2-yn-1-ylamino)propane-3,2-diyl))diacrylamide (**DFC**) in MeOD.

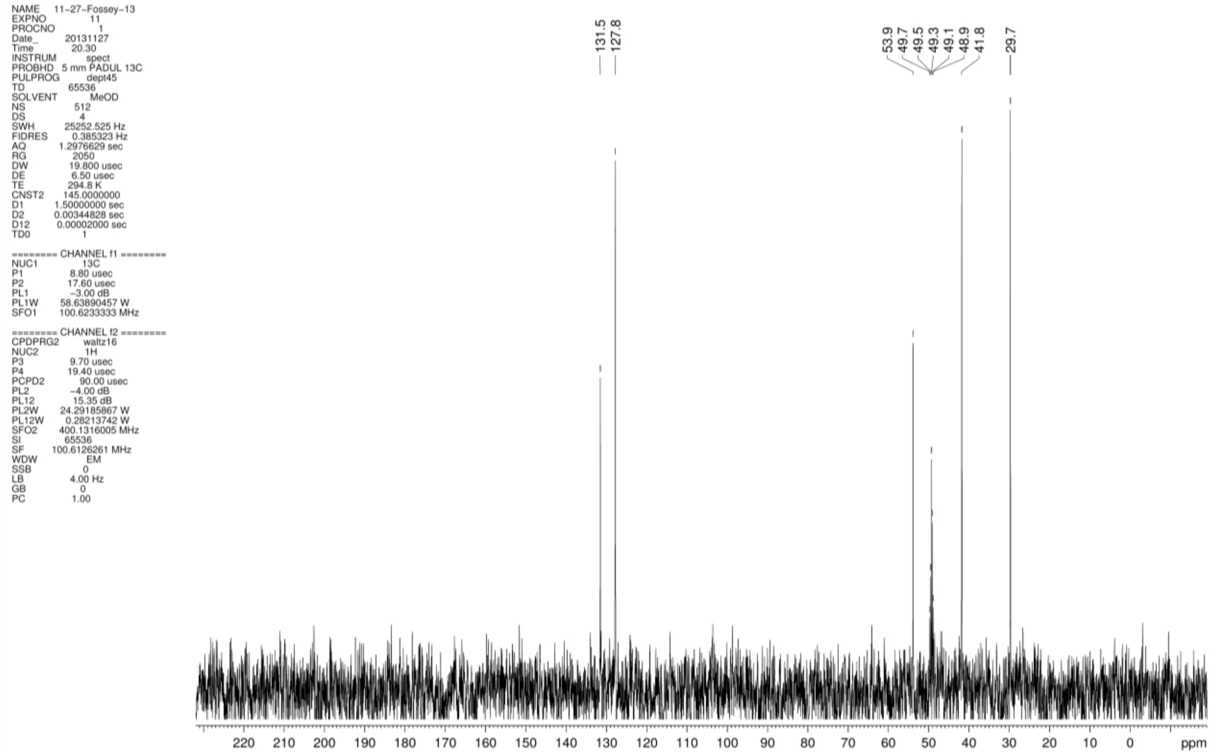

Figure S 15 ^{13}C *N*,*N*'-((2*R*,2'*R*)-disulfanediylbis(1-oxo-1-(prop-2-yn-1-ylamino)propane-3,2-diyl))diacrylamide (**DFC**), in MeOD.

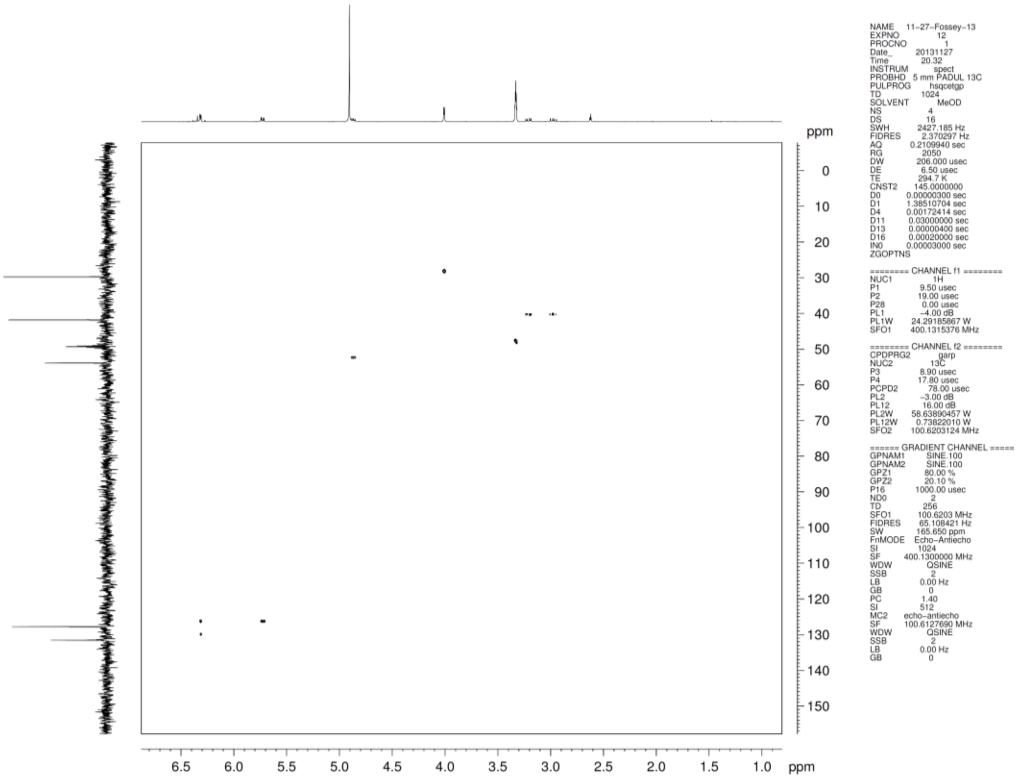

Figure S 16 HSQC spectra of *N*,*N*'-((2*R*,2'*R*)-disulfanediylbis(1-oxo-1-(prop-2-yn-1-ylamino)propane-3,2-diyl))diacrylamide (**DFC**) in MeOD.

5. Surface Modification and Characterisation

5.1 Crosslinking between the DFC SAM and AM-BA

Self-assembled monolayers (SAMs) of **DFC** were placed in an aqueous solution of AM-BA (1 mM, 1 mL) (Sigma Aldrich, UK) which also contained 1% (v/v) tetramethylethylenediamine (TEMED), to which 100 μL of ammonium per sulphate was added (40 mg/mL). The resulting solution was allowed to react for 15 minutes. The modified gold surfaces were subsequently removed from this solution, rinsed for one minute with UHQ water and dried under a stream of argon.

5.2 O-(2-Azidoethyl)heptaethylene glycol (Az-OEG) immobilisation on the DFC SAM via a copper catalysed azide alkyne cycloaddition (Cu-AACA)

An aqueous solution of Az-OEG (5 mM, 1.2 mL) (Sigma Aldrich, UK) was mixed with a copper sulfate (50 μL of a 40 mM) aqueous solution and a sodium ascorbate (50 μL of a 100 mM) solution. SAMs of **DFC** were placed in the Cu-AACA reaction solutions and allowed to react for between 0.5 to 24 hours. After reaction, the gold modified surfaces were removed from Cu-AACA reaction solution and rinsed well with UHQ water and sonicated in ethylenediaminetetraacetic acid (EDTA) solution (0.1 mM) to remove any residual copper.

5.3 Characterization of SAMs and molecularly imprinted surfaces

5.3.1 Contact Angle. Contact angles were determined using a custom-built contact angle apparatus, equipped with a charged coupled device (CCD) KP-M1E/K camera (Hitachi) attached to a personal computer for video capture. The dynamic contact angles were recorded as a micro-syringe was used to quasi-statically add water to or remove water from the drop. The drop was shown as a live video image on the PC screen and the acquisition rate was four frames per second. FTA Video Analysis software v1.96 (First Ten Angstroms) was used for the analysis of the contact angle of a droplet of UHQ water at the three-phase intersection. The averages and standard errors of contact angles were determined from five different measurements made for each type of SAM.

5.3.2 Ellipsometry. The thickness of the deposited monolayers was determined by spectroscopic ellipsometry. A Jobin-Yvon UVISEL ellipsometer with a xenon light source was used for the measurements. The angle of incidence was fixed at 70°. A wavelength range of 280–820 nm was used. DeltaPsi software was employed to determine the thickness values and the calculations were

based on a three-phase ambient/SAM/Au model, in which the SAM was assumed to be isotropic and assigned a refractive index of 1.50. The thickness reported is the average and standard error of six measurements taken on each SAM.

5.3.3 X-Ray photoelectron spectroscopy (XPS). Elemental composition of the SAMs were analysed using an Escalab 250 system (Thermo VG Scientific) operating with Avantage v1.85 software under a pressure of ~ 5 x 10^{-9} mbar. An Al Kα X-ray source was used, which provided a monochromatic X-ray beam with incident energy of 1486.68 eV. A circular spot of size ~ 0.2 mm^2 was employed. The samples were attached onto a stainless steel holder using double-sided carbon sticky tape (Shintron tape). In order to minimise charge retention on the sample, the samples were clipped onto the holder using stainless steel or Cu clips. The clips provided a link between the sample and the sample holder for electrons to flow, which the glass substrate inhibits. Low resolution survey spectra were obtained using a pass energy of 150 eV over a binding energy range of 0 eV to 1250 eV obtained using 1 eV increments. The spectra recorded were an average of three scans. The high resolution spectra were obtained using a pass energy of 20 eV and 0.1 eV increments over a binding energy range of 20–30 eV, centred on the binding energy of the electron environment being studied. A dwell time of 50 ms was employed between each binding energy increment. The spectra recorded were an average of between 5-250 scans (N (1s) = 100, Au (4f) =5, S (2p) = 150, B (1s) = 250, O (1s) = 50, C (1s) = 50). Sensitivity factors used in this study were: N (1s), 1.8; Au (4f), 17.1; S (2p), 1.68; B (1s), 0.486; O (1s), 2.93; C (1s), 1.0.

5.4 Contact angle and ellipsometry analysis of the DFC SAM and grafted AM-BA and Az-OEG on the DFC SAM

The formation of the **DFC** SAM was analysed by means of water advancing (θ_{Adv}) and receding (θ_{Rec}) contact angles and ellipsometry (Table S1). An advancing contact angle of 65° was found for the **DFC** SAM, noting that the hysteresis (θ_{Adv} -θ_{Rec}) value of 18° suggests that the **DFC** SAM is not densely packed. Grafting of either AM-BA or Az-OEG on the **DFC** SAM brought about a change in the wettability of the surfaces, reducing the advancing contact angle from 65° on the **DFC** SAMs to 40° and 56°, respectively. These advancing contact angles are in line with values previously reported for BA-[1] and OEG-terminated SAMs.[2] These results show that the **DFC** SAMs have both functional groups (i.e. alkene and alkyne) accessible to participate in surface reactions. Furthermore, the grafting of the AM-BA and Az-OEG has led to a considerable reduction in contact angle hysteresis from 18° to 9°

and 6°, respectively. Thus, the grafted AM-BA and Az-OEG **DFC** SAMs exhibit a more densely packed structure as compared to that of the **DFC** SAMs, providing indication that both grafting reactions occurred in high yield. Ellipsometry data is also consistent with a high grafting efficiency, with the thickness of the surfaces increasing from 0.42 nm to 1.95 nm and after the AM-BA and Az-OEG glycol were grafted on the **DFC** SAMs, respectively (Table S1). The ellipsometric thickness of the **DFC** SAMs and grafted AM-BA and Az-OEG **DFC** SAMs is less than the theoretical molecular length of the molecules. This discrepancy, between molecular length and SAM thickness, is expected, in agreement with the literature, and it is ascribed to both the tilt angle and density of the SAM surfactants.[3, 4]

Table S1. Advancing and receding water contact angles and ellipsometric thickness for the SAM and grafted AM-BA and Az-OEG on the **DFC** SAM. The theoretical molecular lengths were derived from ChemBio3D Ultra 12.0 in which the molecules were in fully extended conformations.

**SAM**	**Contact Angle (°)**		**Thickness (nm)**	
	Adv.	*Rec.*	*Theor.*	*Exp.*
**DFC**	65 ± 1	47 ± 4	0.91	0.42 ± 0.3
**AM-BA DFC**	40 ± 4	31 ± 3	1.14	0.92 ± 0.3
**Az-OEG DFC**	56 ± 3	50 ± 6	2.90	1.95 ± 0.1

5.5 XPS analysis of the DFC SAM and grafted AM-BA and Az-OEG on the DFC SAM

5.5.1 DFC SAM

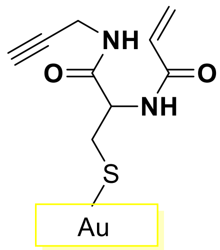

Figure S17 Expected structure for the DFC SAM.

XPS survey spectrum revealed the presence of the elemental species S, N, C, O and Au on the DFC SAM surface (Figure S17). High resolution spectra of S 2p, N 1s and C 2s were acquired in order to unambiguously demonstrate the presence of the DFC SAM on the gold surface (Figure S18). S, N, and C elements were observed in ratios close to those predicted by the molecular structure of the DFC molecule (Table S2), which is consistent with the successful formation of SAMs of the DFC compound.

Table S2: Expected and measured elemental ratios observed for the DFC SAM surfaces, as determined by XPS.

	Expected Ratio	Measured Ratio
**C/S**	9	10.7
**N/S**	2	2.0

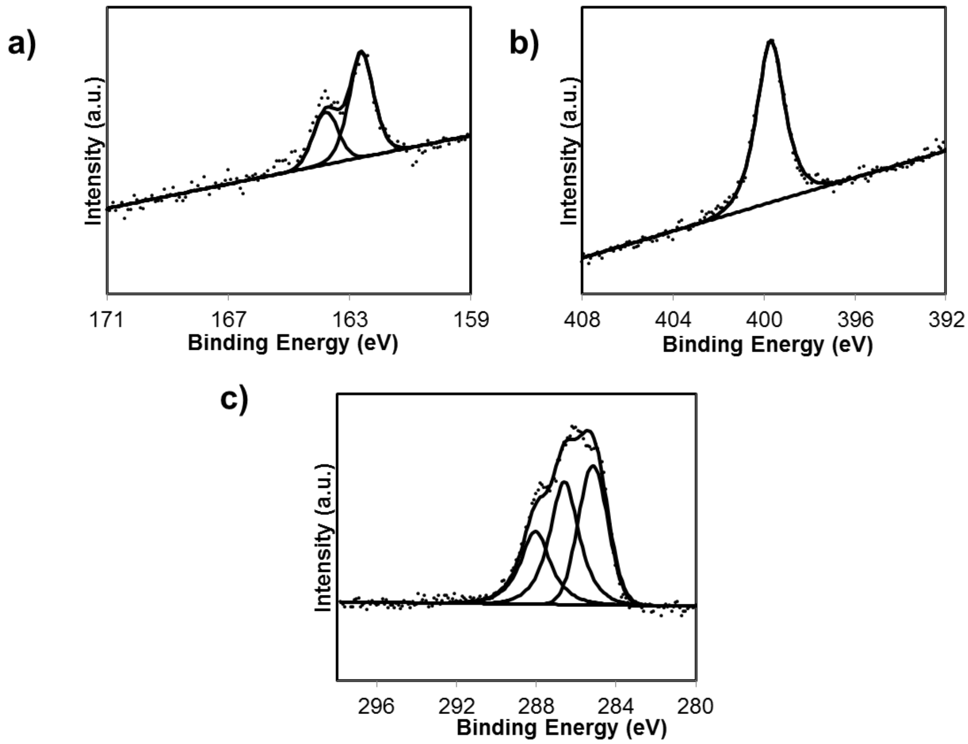

Figure S18 XPS spectra of the a) S 2p, b) N 1s and c) C 1s peak regions of DFC SAMs.

The S 2p spectrum (Figure S18a) consists of a doublet peak at 162.1 eV (S $2p_{3/2}$) and 163.3 eV (S $2p_{1/2}$), indicating that the sulphur is chemisorbed on the gold surface.[5] The N 1s spectrum (Figure S18b) can be assigned to a single peak centred at 399.7 eV, which can be ascribed to the amide groups in the DFC molecule. The C 1s spectrum (Figure S19c) can be resoluted into three peaks,

which is consistent with the structure of the DFC compound. The peak at 285.2 eV is attributed to C-C bonds,[6] while the peak at 286.6 eV corresponds to C 1s of the two binding environments of C-S and C-N. The third and smaller peak (288.0 eV) is assigned to the C 1s photoelectron of the carbonyl moiety, C=O.[7] The ratio of the three carbon peaks C-C:C-S/C-N:C=O is 1.8:1.6:1, which is in good agreement with the expected ratio of 2:1.5:1.

5.5.2 Grafted AM-BA on the DFC SAM

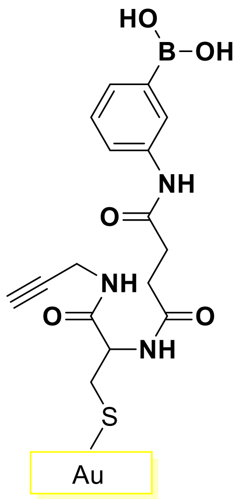

Figure S19 Expected structure for the grafted AM-BA on the DFC SAM.

High-resolution XPS spectra of S 2p, N 1s, C 2s and B 1s confirmed the grafting of the AM-BA on the DFC SAM (Figure S19). S, N, C and B elements were observed in ratios close to those expected for a 1:1 stoichiometric reaction (Table S3), illustrating that the AM-BA can be incorporated on the DFC SAM with quantitative grafting efficiency.

Table S3: Expected and measured elemental ratios observed for the AM-BA modified DFC surfaces, as determined by XPS.

Element	Expected Ratio	Measured Ratio
**C/S**	20	21.4
**N/S**	3	3.1
**B/S**	1	1.1

The S 2p spectrum (Figure S20a) consists of a doublet peak at 162.1 eV (S $2p_{3/2}$) and 163.3 eV (S $2p_{1/2}$), indicating that the sulphur is chemisorbed on the gold surface.[5] The N 1s spectrum (Figure S20b) contained a single peak centred at 400.4 eV, which is attributed to the amide moieties.[8] The C 1s spectrum (Figure S20c) can be resolved into three peaks, which are attributed to five different carbon binding environments. The peak at 285.1 eV is attributed to C-C bonds[6], while the peak at 286.5 eV corresponds to C 1s of the three binding environments of C-S, C-N and C-B.[6] The third and smaller peak (288.4 eV) is assigned to the C 1s photoelectron of the carbonyl moiety, C=O.[6] The ratio of the three carbon peaks C-C:C-S/C-N/C-B:C=O is 3:1.4:1, which is in good agreement with the expected ratio of 2.7:1.6:1. The B 1s spectrum displays a peak at 192 eV, in good agreement with the values reported for other boronic acid derivatives. [9]

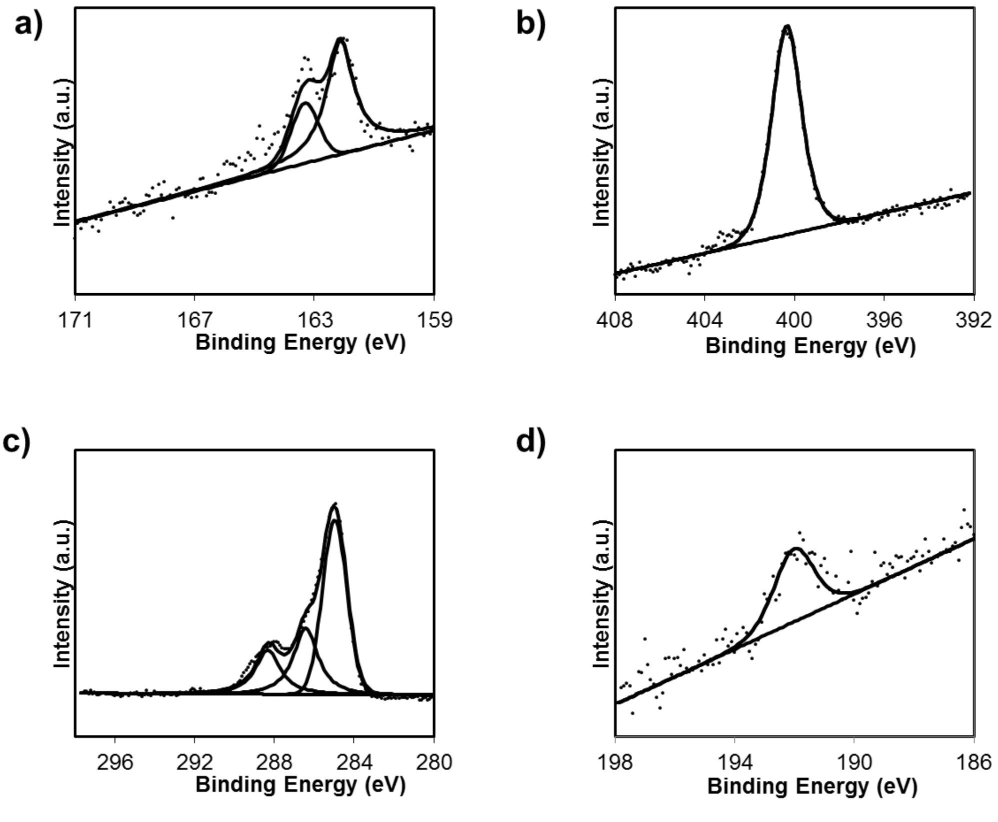

Figure S20 XPS spectra of the a) S 2p, b) N 1s, c) C 1s and d) B 1s peak regions of AM-BA modified DFC surfaces.

5.5.3 Grafted Az-OEG on the DFC SAM

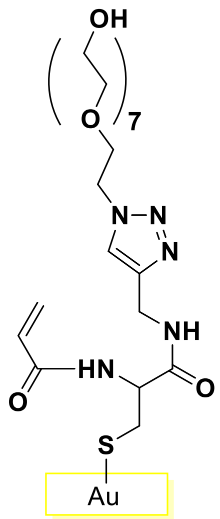

Figure S21 Expected structure for the DFC SAM modified via copper catalysed azide alkyne cycloaddition reaction with Az-OEG.

High-resolution XPS spectra of S 2p, N 1s and C 2s confirmed the success of the DFC SAM modification via copper catalysed azide alkyne cycloaddition (CuCAA) reaction with Az-OEG (Figures S21 and S22). The S, N and C elements were observed in the ratios expected (Table S4), and were consistent with a near quantitative yield for the surface CuCAA reaction.

Table S4: Expected and measured elemental ratios observed for the grafted Az-OEG on the DFC SAM, as determined by XPS.

Element	Expected Ratio	Measured Ratio
N/S	5	4.8
C/S	25	24.9

The S 2p spectrum (Figure S22a) consists of a doublet peak at 162.3 eV (S $2p_{3/2}$) and 163.5 eV (S $2p_{1/2}$), indicating that the sulphur is chemisorbed on the gold surface.[5] The N 1s spectrum (Figure S22b) can be resolved into two peaks at 399.5 eV (N=N and NH-C=O) and 400.6 eV (C-N).[10, 11] No peaks were observed at higher binding energies, such as those which may be produced by the electron deficient nitrogen present in the azide starting material.[12] The ratio of the two nitrogen peaks N=N/NH-C=O:C-N is 3.9:1, which in good agreement with the expected ratio of 4:1.

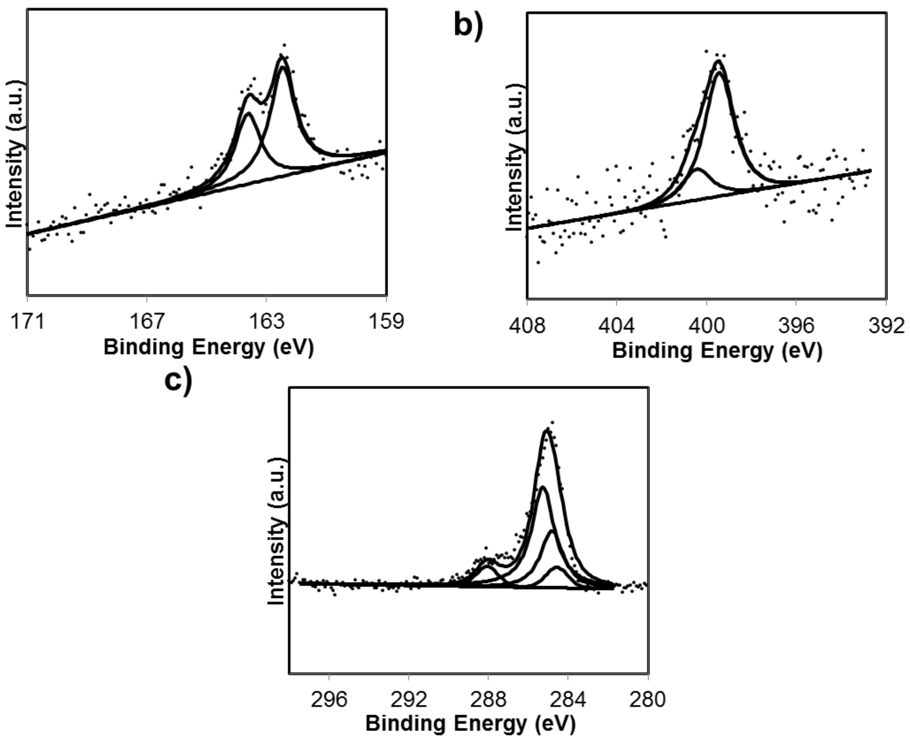

Figure S22 XPS spectra of the a) S 2p, b) N 1s and c) C 1s peak regions of Az-OEG modified DFC surfaces.

The C 1s spectrum (Figure S22c) can be resolved into four peaks. The peak at 284.6 eV is attributed to C-C bonds,[6] while the peak at 284.8 eV corresponds to C 1s of the three binding environments of C-S, C-N and C-OH.[6] The third peak centred at 285.3 eV is attributed to the C-O bonds from the OEG moieties. The fourth peak (288.1 eV) is assigned to the C 1s photoelectron of the carbonyl moiety, C=O.[7] The C-C:C-S/C-N/C-OH:C-O:C=O ratio of these peaks was found to be 1.9:6.8:13.6:2, which is close to the expected ratio of the carbon environments (2:7:14:2). Taken together with the analysis of the N 1s spectrum, this finding is consistent with a near quantitative CuCAA reaction of the DFC SAM with Az-OEG moieties.

6. SPR analysis of binding of proteins on DFC SAMs grafted with Az-OEG

Table S5: SPR responses at equilibrium of DFC SAMs grafted with Az-OEG to different proteins at a concentration of 650 nM.

	Lysozyme	RNAse A	BSA	RNAse B	α1-AT	HRP	α1-AGP
**SPR Response (Response units)**	22.2 ± 5.9	21.9 ± 6.2	21.4 ± 1.2	21.2 ± 7.6	4.3 ± 10.7	10.5 ± 13.1	3.8 ± 2.3

7. References

1. C. Vahlberg, M. Linares, P. Norman and K. Uvdal, *J. Phys. Chem. C*, 2012, **116**, 796-806.
2. A. Stephenson-Brown, H. C. Wang, P. Iqbal, J. A. Preece, Y. T. Long, J. S. Fossey, T. D. James and P. M. Mendes, *Analyst*, 2013, **138**, 7140-7145.
3. A. Ulman, *Chem. Rev.*, 1996, **96**, 1533-1554.
4. H. L. Zhang, J. Zhang, H. Y. Li, Z. F. Liu and H. L. Li, *Mat. Sci. Eng. C - Bio S.*, 1999, **8-9**, 179-185.
5. T. Weidner, F. Bretthauer, N. Ballav, H. Motschmann, H. Orendi, C. Bruhn, U. Siemeling and M. Zharnikov, *Langmuir*, 2008, **24**, 11691-11700.
6. J. F. Moulder, W. F. Stickle, P. E. Sobol and K. D. Bomben, *Handbook of X-ray Photoelectron Spectroscopy*, Perkin-Elmer Corp, Eden Prairie, MN, USA, 1992.
7. G. Dodero, L. De Michieli, O. Cavalleri, R. Rolandi, L. Oliveri, A. Daccà and R. Parodi, *Colloids Surf., A*, 2000, **175**, 121-128.
8. R. J. J. Jansen and H. van Bekkum, *Carbon*, 1995, **33**, 1021-1027.
9. W. Wu, H. Zhu, L. Fan, D. Liu, R. Renneberg and S. Yang, *Chem. Comm.*, 2007, 2345.
10. N. Maalouli, A. Barras, A. Siriwardena, M. Bouazaoui, R. Boukherroub and S. Szunerits, *Analyst*, 2013, **138**, 805-812.
11. S. Ciampi, E. Luais, M. James, M. H. Choudhury, N. A. Darwish and J. J. Gooding, *Phys. Chem. Chem. Phys.*, 2014, **16**, 8003-8011.
12. S. Ciampi, M. James, M. H. Choudhury, N. A. Darwish and J. J. Gooding, *Phys. Chem. Chem. Phys.*, 2013, **15**, 9879-9890.
